# Benzofuran Derivatives with Antimicrobial and Anti-Inflammatory Activities from *Penicillium crustosum* SCNU-F0046

**DOI:** 10.3390/ijms26167861

**Published:** 2025-08-14

**Authors:** Chen Chen, Jinbi Kang, Ruiqi Zhang, Hao Jia, Zirong Lin, Zhengming Liu, Rongrong Liu, Xinyi Zou, Yuhua Long

**Affiliations:** Guangzhou Key Laboratory of Analytical Chemistry for Biomedicine, School of Chemistry, South China Normal University, Guangzhou 510006, China; chenchen2021@m.scnu.edu.cn (C.C.); 2024022687@m.scnu.edu.cn (J.K.); rickie@m.scnu.edu.cn (R.Z.); haojia@m.scnu.edu.cn (H.J.); linzirong@m.scnu.edu.cn (Z.L.); 2023022616@m.scnu.edu.cn (Z.L.); rongrongliu@m.scnu.edu.cn (R.L.); 2024022764@m.scnu.edu.cn (X.Z.)

**Keywords:** *Penicillium crustosum*, benzofurans, anti-inflammatory activity, antimicrobial activity

## Abstract

A chemical investigation on the marine-derived fungus *Penicillium crustosum* SCNU-F0046 resulted in the isolation and characterization of four new benzofurans (**1**, **2**, **5**, **6**) and four known analogues (**3**, **4**, **7**, **8**). Their structures were elucidated by a combination of mass, NMR spectroscopy, electronic circular dichroism (ECD) calculations and X-ray crystallographic analyses. The antimicrobial experiments disclosed compound **1** exhibited moderate antibacterial activity, while compound **6** showed antifungal activity. In addition, the anti-inflammatory activity of aza-benzofuran compounds (**1**–**4**) was also evaluated. Bioassays revealed that compounds **1** and **4** exhibited anti-inflammatory activity by inhibiting nitric oxide release without cytotoxicity in lipopolysaccharide (LPS)-stimulated RAW 264.7 mouse macrophages with IC_50_ values of 17.3 and 16.5 μM, respectively. The docking study revealed that compounds **1** and **4** exhibited an ideal fit within the active site of the murine inducible nitric oxide synthase (iNOS), establishing characteristic hydrogen bonds.

## 1. Introduction

Natural products play an increasingly vital role in drug discovery due to their great chemical diversity. The marine environment covers 71% of the Earth’s surface and comprises 50–80% of global biodiversity, which translates into enormous chemical diversity [1,2]. Every year, hundreds of novel compounds are isolated and identified [3,4]. In the past decade, several therapeutic agents, such as the anti-tumor drug trabectedin [5], the analgesic ziconotide [6], and brefeldin A (a Golgi-disruptor and Arf-GEFs inhibitor) [7], have been developed from these marine natural products, which exhibit structural diversity and biological activities [8]. Compared to terrestrial plants and non-marine microorganisms, marine-derived fungi can be considered the most recent source of bioactive natural products [9,10].

Mangroves are a special marine ecosystem that grows at the land–sea interface in tropical and subtropical regions, characterized by high salinity, low oxygen, tidal gradients, high temperatures, and intense light. These conditions support an active microbial community [11,12,13]. Among marine fungal communities, mangrove-associated fungi constitute the second-largest ecological group [14]. Microbes have developed effective mechanisms to adapt their metabolic pathways, leading to the production of unique chemicals and significant biological diversity. This makes mangrove-associated fungi a rich source of unique chemical structures and diverse pharmacological activities [11,15]. The secondary metabolites from mangrove fungi mainly include polyketides, terpenoids, alkaloids, and peptides [11]. In addition, these metabolites exhibit a wide range of pharmacological activities, such as antioxidative activity [16], cytotoxic activity [17], antifungal activity [18], anti-inflammatory activity [19], and α-glucosidase inhibitory activity [20], among others. These findings indicate that mangrove endophytic fungi are valuable sources of novel and active secondary metabolites.

**Figure 1:**
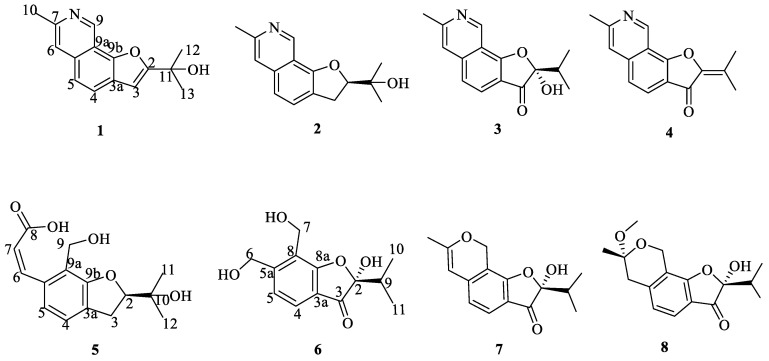
Structures of compounds **1**–**8**.

As a part of our continuing search for bioactive compounds from marine-derived fungi, a chemical study on the culture broth of *Penicillium crustosum* SCNU-F0046 was carried out [21,22,23]. As a result, four new benzofurans derivatives (**1**, **2**, **5**, **6**) and four known analogues (**3**, **4**, **7**, **8**) were isolated from the EtOAC extract after its scale-up fermentation (Figure 1).

## 2. Results and Discussion

### 2.1. Structures of Compounds ***1***, ***2***, ***5*** and ***6***

**Figure 2:**
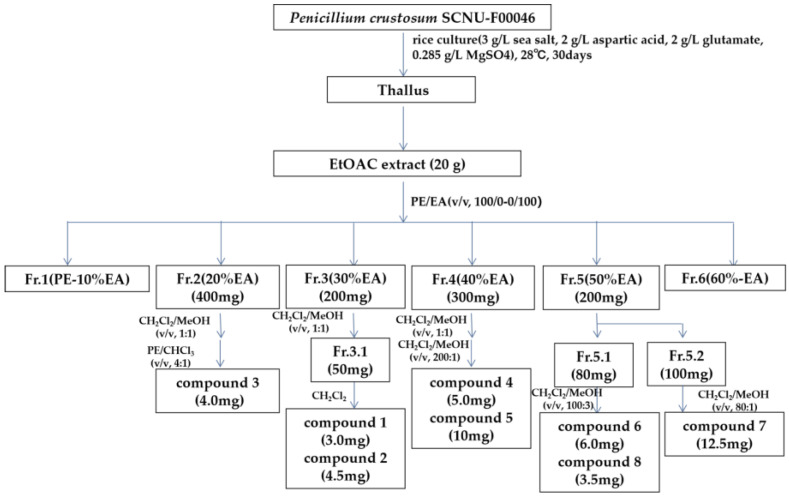
Isolation flow chart of strain *Penicillium crustosum* SCNU-F00046 on 3 g/L sea salt, 2 g/L aspartic acid, 2 g/L glutamate, 0.285 g/L MgSO_4_ rice solid medium.

The EtOAc extract of marine-derived fungus *Penicillium crustosum* SCNU-F0046 was performed on repeated silica gel and Sephadex LH-20 column chromatography, followed by semi-preparative HPLC to afford four new benzofurans along with four known benzofurans (Figure 2).

**Figure 3:**
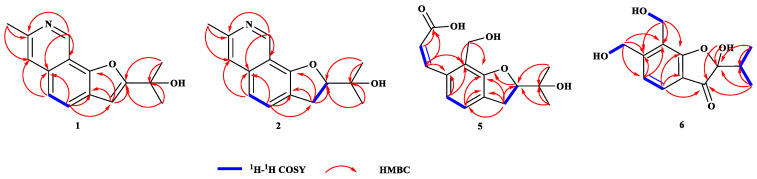
Key COSY, HMBC correlations of compound **1**, **2**, **5** and **6**.

**Figure 4:**
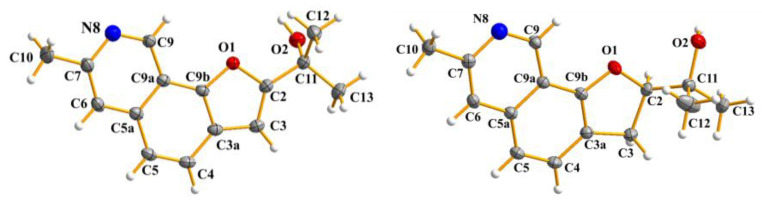
The single-crystal X-ray crystallography structures of compounds **1** and **2**.

Compound **1** was obtained as light-yellowish solid and consequently crystallized from CH_2_Cl_2_/MeOH (*v*/*v*, 1:1) as light-yellowish needle-like crystals. Its molecular formula was determined to be C_15_H_16_NO_2_ from the HRESIMS equimolecular ion peak at *m*/*z* 242.11752 [M + H]^+^ (Figure S1), which was consistent with nine degrees of unsaturation. The ^1^H NMR spectrum (Figure S2) of **1** showed two aromatic protons at *δ*H 7.76 (d, *J* = 8.46 Hz, H-4), 7.51 (d, *J* = 8.46 Hz, H-5); two monosubstituted pyridine moieties at *δ*H 9.65 (s, H-9), 7.55 (s, H-6); one olefinic methine at *δ*H 6.75 (s, H-3); three methyl singlets at *δ*H 2.74 (s, H-10), 1.78 (s, H-12), and 1.78 (s, H-13) (Table 1). The ^13^C NMR spectrum (Figure S3) together with the HSQC spectrum (Figure S4) revealed the presence of eleven olefinic carbons (*δ*C 163.5, 151.3, 149.5, 144.5, 134.8, 124.8, 124.7, 120.2, 119.2, 115.2, 101.7), oxygenated non-protonated carbon (*δ*C 69.4), three methyls (*δ*C 29.0, 29.0, 24.2). The ^1^H-^1^H COSY relationship of H-5 with H-4 is shown in Figure S6. The ^1^H and ^13^C NMR spectroscopic data of compound **1** were closely related to those of TMC-20 novel derivative 1 [24], except for the downfield shifts of C-2, C-3, and C-3a, rom *δ*C 90.8, 30.4, and 122.0 in TMC-120 novel derivative 1 to *δ*C 163.48, 101.70, and 149.49 in compound **1**, respectively, indicating the existence of a double bond between C-2 and C-3. These observations were confirmed by the HMBC (Figure S5) correlations of H-3 to C-2, C-3a and C-4 (Figure 3). The structure of **1** was further confirmed by single-crystal X-ray diffraction (Figure 4).

Compound **2** was assigned the molecular formula C_15_H_18_NO_2_ on the basis of HRESIMS, indicating eight degrees of unsaturation (Figure S7). The ^1^H NMR (Table 2) spectra of **2** revealed the presence of three methyls at *δ*H 2.48 (3H, s), 1.54 (3H, s), 1.29 (3H, s) and four olefinic protons at *δ*H 9.26 (1H, s), 7.42 (1H, d, *J* = 8.16 Hz), 7.19 (1H, s), 7.08 (1H, d, *J* = 8.16) (Figure S8). Analysis of the ^13^C NMR data together with the HSQC spectrum revealed 15 carbon resonances and displayed the presence of one methylene (*δ*C 30.8), three methyls (*δ*C 26.7, 1.54, 1.29), six quaternary carbons (*δ*C 155.7, 150.4, 136.6, 122.0, 113.9, 71.4), four olefinic carbons (*δ*C 145.8, 127.8, 120.2, 118.4) (Figures S9 and S10). The ^1^H and ^13^C NMR data of **2** were consistent with those previously reported for the TMC-20 novel derivative 1 [24] isolated from *Aspergillus ustus TC* 1118, with essentially the same chemical shifts and the corresponding coupling patterns. In addition, the COSY (Figure S12) and HMBC (Figure S11) relationships of compound **2** are also consistent with those of compound TMC-20 novel derivative 1 (Figure 3). However, the rotation value of **2** was opposite to that the TMC-20 novel derivative 1, revealing that these two compounds were the enantiomers. A single-crystal X-ray diffraction analysis of **2** with the Flack parameter [0.17 (4)] enabled us to further confirm its absolute configuration as *S* (Figure 4).

**Figure 5:**
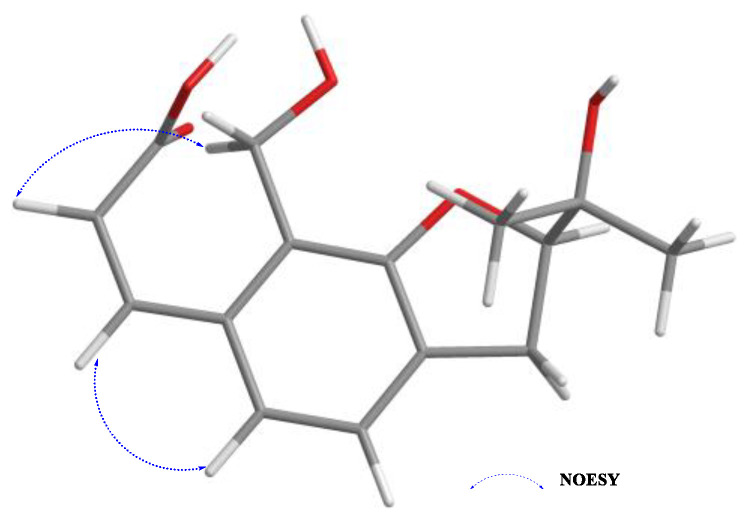
NOESY correlations of **5**.

**Figure 6:**
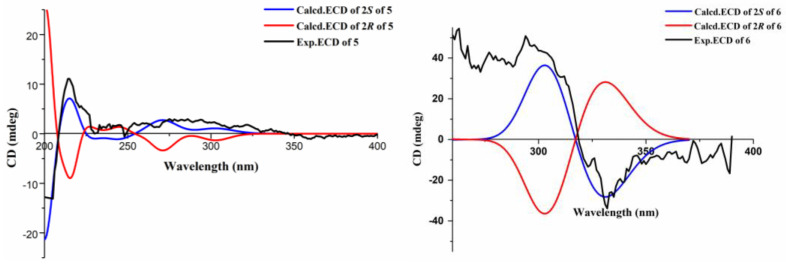
Calculated and experimental ECD spectra of **5** and **6**.

Compound **5** was obtained as a white powder; its molecular formula, C_15_H_18_O_5_, was assigned according to the HRESIMS data in conjunction with NMR analyses (Figure S13). The ^1^H NMR spectrum (Figure S14) of compound **5** showed the existence of a 1, 2, 3, 4-tetrasubstituted benzene ring, with the signals at *δ*H 7.21 (d, *J* = 7.8 Hz, H-4) and 7.17 (d, *J* = 7.8 Hz, H-5). In addition to this, the ^1^H NMR spectrum of **5** also showed two olefinic methines at *δ*H 8.08 (d, *J* = 15.9 Hz, H-8), 6.41 (d, *J* = 15.9 Hz, H-7), two methyls at *δ*H 1.33 (s, H-11), 1.24 (s, H-12), two methylenes at *δ*H 4.75 (s, H-9), 3.25 (M, H-3), and one methine group at *δ*H 4.64 (t, *J* = 9.1 Hz, H-2) (Table 3). The ^13^C NMR data and HSQC spectrum data of **5** contained 15 carbons, including 1 carbonyl (*δ*C 170.84), 8 olefinic carbons (*δ*C 160.0, 143.5, 135.24, 130.92, 125.49, 121.83, 120.61 and 120.05), 2 methyls (*δ*C 25.7, 24.8), 2 methylenes (*δ*C 55.4, 31.6), and 1 methine (*δ*C 90.5) (Figures S15 and S16). The ^1^H and ^13^C NMR spectroscopic data of compound **5** exhibited similarities to Asperspin A [25], except for the methoxy group (*δ*C 58.5) and one methylene (*δ*C 73.3) present in Asperspin A, which was substituted by a carboxyl group (*δ*C 170.8) in compound **5**, which was verified by the ^1^H−^1^H COSY (Figure S18) correlations of H-6 with H-7 and the HMBC (Figure S17) correlations from H-6 (*δ*H 6.4) to C-8 (*δ*C 170.8), C-9a (*δ*C 55.4) and H-7 (*δ*H 8.08) to C-8 (*δ*C 170.8) (Figure 3). The configuration of the double bond between C-6 and C-7 was assigned as *E* form according to the NOESY correlations (Figure S19) of H-6 with H-5 and H-7 with H-9, as well as a large coupling constant of 15.9 Hz (Figure 5). The absolute configuration of the C-2 chiral center in compound **5** was ascertained to be *R*. This determination was made by comparing the optical rotation value of compound **5**, which was ([α]_25_ᴰ = −18.8), with the optical rotation value of the alcohol [(−) −V] that has been previously reported in the relevant literature. Moreover, this conclusion was further confirmed by electronic circular dichroism (ECD) calculations (Figure 6).

Compound **6** was isolated as a white powder. It showed an HRESIMS peak at *m*/**z** 251.0927 [M–H]^−^ (Figure S20), indicating a molecular formula of C_13_H_15_O_5_. A careful comparison of its ^1^H, ^13^C NMR spectra and HSQC spectra (Figures S21–S23) with those of Asperisocoumarin H indicated that compound **6** also shared the same 3-coumaranone skeleton as Asperisocoumarin H [26]; the most significant difference between compound **6** and Asperisocoumarin H is the structural characterization of C-6 and C-7 (Table 4). In Asperisocoumarin H, a pyran ring is formed at these positions. In contrast, in compound **6**, C-6 and C-7 are each bound to a hydroxyl group. These aspects can be convincingly substantiated by means of the HMBC (Figure S24) correlations of H-6 (*δ*H 4.74) to C-5a (*δ*C 153.7), C-5 (*δ*C 112.0), C-8 (*δ*C 122.8), and H-7 (*δ*H 4.56) to C-5a (*δ*C 153.7), C-8 (*δ*C 122.8), C-8a (*δ*C 168.8) (Figure 3), as well as the ^1^H-^1^HCOSY relationship of OH-6 with H-6 and OH-7 with H-7 (Figure S25). The configuration of stereocenters was assigned as 2*S,* as the experimental ECD curve showed good agreement with the curves (Figure 6).

The other known compounds were identified as TMC-120 C (**3**), TMC-120 B (**4**) [27], Penicisochroman H (**7**) [28], Penicisochroman F (**8**) [28] via NMR data analysis and a comparison with the reported spectroscopic data.

### 2.2. Antimicrobial Assay

The minimum inhibitory concentration values (MIC) of all new compounds **1**, **2**, **5** and **6** were determined within the concentration range of 0.78–100 μg/mL by the 96-well plate broth dilution method. As shown in Table 5, compound **1** had moderate antibacterial activity against Salmonella typhimurium, Escherichia coli and Staphylococcus aureus, with MIC values of 12.5 μg/mL, 25 μg/mL and 12.5 μg/mL, respectively; compound **2** had weak antibacterial activity against Staphylococcus aureus (MIC = 25 μg/mL). In comparison with the positive control ciprofloxacin, both compound 1 and compound **2** exhibited lower activity. Compounds **5** and **6** showed antifungal effects against Penicillium italicum and Colletotrichum musae, with MIC values of 12.5 μg/mL and 12.5–25 μg/mL, respectively. Therefore, for bacterial inhibition activity, aza-benzofuran compounds exhibit better activity than oxa-benzofuran compounds. In contrast, for fungal inhibition activity, aza-benzofuran compounds show lower activity than oxa-benzofuran compounds. Structural–activity relationships indicate that aza-benzofuran compounds exhibit higher activity than their oxa-benzofuran counterparts; this may be attributed to their enhanced lipophilicity and the ability to carry a positive charge, which facilitates electrostatic interactions with bacterial membranes, disrupting their integrity. Additionally, compound 1 shows greater activity than compound **2**, suggesting that the conjugated system in compound 1 expands when the C-2 and C-3 positions form double bonds, thereby enhancing hydrophobicity. In terms of antifungal activity, when compound **5** and **6** form hydroxyl groups at position C-6, their polarity decreases, making them more susceptible to penetrating fungal cell membranes through hydrophobic mechanisms.

### 2.3. Anti-Inflammatory Assay

The anti-inflammatory activities of compounds **1**–**4** were evaluated by assessing the inhibition of nitric oxide (NO) production in lipopolysaccharide (LPS)-stimulated murine macrophage RAW 264.7 cells. As shown in Table 6, compounds **1** and **3** exhibited significant inhibitory effects with IC_50_ values of 17.31 μM and 16.5 μM, respectively, compared to celecoxib (positive control, IC_50_ = 32.1 ± 1.7 μM), while compound **2** (IC_50_ = 31.5 ± 2.3 μM) and compound **4** (IC_50_ = 42.8 ± 4.7 μM) displayed moderate activity compared to celecoxib (positive control, IC_50_ = 32.1 ± 1.7 μM). Cytotoxicity assessments using the MTT assay (Table 3) revealed that compounds **1**–**4** did not induce significant cytotoxicity at concentrations up to 50 μM. Structure–activity relationship (SAR) analysis indicated that the presence of a double bond between C-2 and C-3 in compound **1** conferred superior anti-inflammatory activity compared to the single-bond configuration in compound **2**, as supported by their respective IC_50_ values. Furthermore, comparing compounds **3** and **4**, we can conclude that compounds forming double bonds at C-2 and C-11 exhibited potent anti-inflammatory activities. In addition, we also tested the cytotoxicity of compounds **1**–**4,** and cytotoxicity was assessed independently of LPS stimulation. Compounds **1**–**4** displayed weak activity against the RAW264.7 cell line.

### 2.4. Molecular Docking Studies

**Figure 7:**
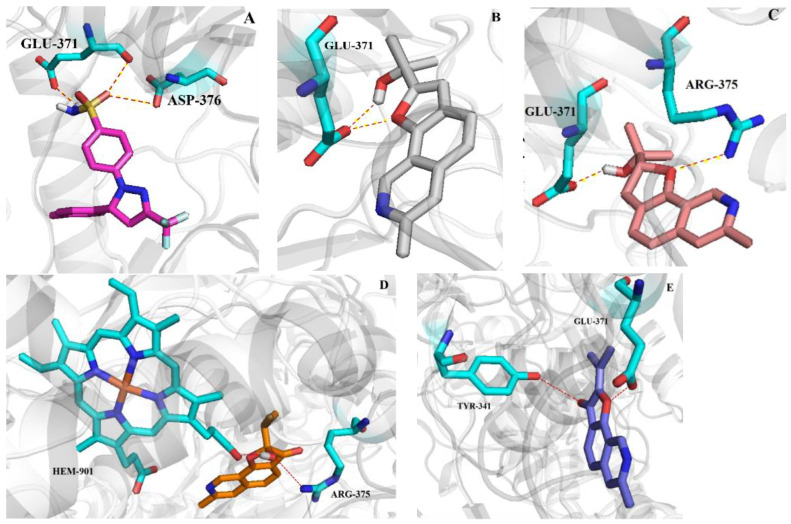
Molecular docking simulations were obtained at the lowest energy conformation, highlighting potential hydrogen contacts of positive drug celecoxib ((**A**), magenta), compound **1** ((**B**), gray), **2** ((**C**), salmon), **3** ((**D**), orange), **4** ((**E**), blue) respectively. For clarity, only interacting residues are labeled. Hydrogen bonding interactions are indicated by dashed lines. These plots were created by Poul.

In order to understand the mechanism of NO inhibition, a molecular docking experiment was performed. The molecular docking study was performed using AUTODOCK 4.4.6 modeling software. We docked the positive drug celecoxib to validate the docking protocol with the crystal structure iNOS (PDB:3E6T [29]). The docking protocol, described in the experimental section, successfully reproduced the bound conformation of ligand celecoxib (positive drug) with a root-mean-square deviation (RMSD) of 0.12 Å to the X-ray structure. The results revealed that the lowest energy of compound **1** (−8.39 kcal/mol), compound **2** (−8.14 kcal/mol) and **4** (−8.42 kcal/mol) was lower than that of positive drug celecoxib (−8.0 kcal/mol), and the lowest energy of compound **3** (−7.36 kcal/mol) was higher than that of the positive drug. Further observations showed celecoxib formed two hydrogen bonds with key amino acid residues GLU-371 and one hydrogen bond with amino acid residues ASP-376 in the iNOS active pocket (Figure 7A). Compound **1** formed two hydrogen bonds with the key amino acid residue GLU-371 through the hydroxyl group and the oxygen atom in the furan ring in the iNOS active pocket (Figure 7B), respectively. Compound **2** formed one hydrogen bond with the key amino acid residue GLU-371 through the hydroxyl group, and one hydrogen bonded with the residue ARG-375 by the oxygen atom in the furan ring in the iNOS active pocket (Figure 7C). Compound **4** formed one hydrogen bond with the key amino acid residue GLU-371 through the oxygen atom in the furan ring, and one hydrogen boned with the residue TYR-341 by carbonyl group in the iNOS active pocket (Figure 7E). Notably, for compound **3**, while the double bond in compound **3** at the **2** position changed to the hydroxyl group, the optimized conformation of **3** was different from that of compound **4** and could not form hydrogen bonds with the key amino acid residues (Figure 7D). As a result, compound **3** has relatively low production inhibition activity (Table 6). Based on the above results, we can speculate that in virtual docking, when the oxygen atom in the furan ring of the compound interacts with key amino acid residues, the compound exhibits better activity.

## 3. Experimental Section

### 3.1. General Experimental Procedures

NMR spectra were measured on Bruker Avance 400 and 500 spectrometers (Bruker BioSpin Corporation, Billerica, MA, USA) at room temperature using tetramethyl silane (TMS) as an internal standard. Optical rotations were determined using an Anton Paar (MCP 300) polarimeter at 25 °C. The high-resolution electrospray ionization mass spectrometry (HRESIMS) was utilized on a quadrupole time-of-flight (Q-TOF) high-resolution mass spectrometer (Waters Corporation, Synapt G2-Si, Milford, MA, USA). UV spectra were measured with a UV-240 spectrophotometer (Shimadzu, Beijing, China). Column chromatography (CC) was carried out on silica gel (200–300 mesh, Qingdao Marine Chemical Factory, Qingdao, China) and Sephadex LH-20 (Amersham Pharmacia, Piscataway, NJ, USA). GF-254 precoated silica gel plates (Qingdao Huang Hai Chemical Group Co., Qingdao, China) were used for thin-layer chromatography.

### 3.2. Fungal Material

The strain *Penicillium crustosum* SCNU-F00046 is a mangrove fungus collected from the Yangjiang Mangrove Nature Reserve in Guangdong Province, China. On 10 September 2019, fresh samples collected from the mangrove wetland reserve were preliminarily rinsed with sterile water, after which the roots, stems, leaves, flowers, fruits and seeds of the plants were separated. Using sterilized tweezers, the plant tissues were sequentially disinfected in 3% sodium hypochlorite solution and 75% ethanol solution, followed by rinsing with sterile water to remove residual solvents. Subsequently, the stems of mangrove were cut into small segments of approximately 0.2 × 0.6 cm and transferred to autoclaved Bengal Rose agar plates. After sealing the plates, they were incubated at room temperature in a sterile environment. When colonies appeared after 2–3 days, they were picked using a flame-sterilized sterile inoculating loop. Endophytic fungal strains were isolated via conventional microbiological protocols. Subsequently, the fungal isolates were numbered and stored in triplicate on PDA slants at 4 °C. DNA amplification and sequencing of the fungus *Penicillium crustosum* were conducted as follows. Approximately 120 mg of fresh fungal mycelia was harvested into 1.5 mL microcentrifuge tubes, and genomic DNA was isolated using the Fungal DNA Kit (D3171, MAGEN, Guangzhou, China, Magen Biotechnology Co., Ltd.) in accordance with the manufacturer’s protocol. PCR amplification was performed in a total volume of 50 μL, comprising 2 μL template DNA, 5 μL 10× reaction buffer, 1 μL dNTPs, 0.5 μL ITS1F, 0.5 μL ITS4 (20 μmol·mL^−1^ each), 0.25 μL Taq polymerase, and nuclease-free water to bring the volume to 50 μL. The amplification conditions were as follows: initial denaturation at 94 °C for 5 min, followed by 32 cycles of denaturation at 94 °C for 50 s, annealing at 52.5 °C for 50 s, and extension at 72 °C for 1 min, with a final extension at 72 °C for 10 min. Subsequently, 5 μL of the amplified product was loaded onto a 1.2% agarose gel prepared in 0.5× TAE buffer, supplemented with 5 μL of 1% (m/v) ethidium bromide per 100 mL of gel. After electrophoresis at 100 V for 40 min, the ~600 bp PCR product band was excised from the gel and purified using a Gel Extraction Kit (cat. no. D2110, MAGEN, Guangzhou, China, Magen Biotechnology Co., Ltd.) according to the manufacturer’s instructions. The purified PCR product was then submitted for sequencing with primer ITS1F (Ruibo Biotechnology, Guangzhou, China). The PCR-amplified sequence is as follows: TCCGTAGGTGAACCTGCGG, TCCTCCGCTTATTGATATGC. The strain was identified according to the ITS rDNA sequence (GenBank accession number PP941960). BLAST+2.14.0 alignment analysis revealed that this strain exhibits the highest sequence similarity (99%) to *Penicillium crustosum*. The voucher strain has been deposited in the College of Chemistry, South China Normal University, Guangzhou, China.

### 3.3. Fermentation and Isolation

The metabolites from the rice culture (3 g/L sea salt, 2 g/L aspartic acid, 2 g/L glutamate, 0.285 g/L MgSO_4_) were extracted with EtOAc. The solution was evaporated to dryness under reduced pressure to afford an EtOAC extract (20 g). The extract was chromatographed on silica gel CC using gradient elution with petroleum ether-EtOAc from 100:0 to 0:100 (*v*/*v*) to give seven fractions (fractions 1–6). Fraction 2 (400 mg) was separated on Sephadex LH-20 CC using CH_2_Cl_2_/MeOH (*v*/*v*, 1:1), followed by silica gel CC eluting with PE/CHCl_3_ (*v*/*v*, 4:1) to yield compound **3** (4.0 mg). Fraction 3 (200 mg) was applied to Sephadex LH-20 CC and eluted with CHCl_3_/MeOH (*v*/*v*, 1:1) to obtain 3 subfractions (fractions 3.1–3.3). Fraction 3.1 (50 mg) was further purified by silica gel CC with CH_2_Cl_2_ to afford compound **1** (3.0 mg) and compound **2** (4.5 mg). Fraction 4 (300 mg) was subjected to Sephadex LH-20 CC (CH_2_Cl_2_/MeOH, *v*/*v*, 1:1) and silica gel glass column eluted with CH_2_Cl_2_/MeOH (*v*/*v*, 200:1) to obtain compound **5** (10 mg) and compound **4** (5 mg). Fraction 5 (200 mg) was subjected to silica gel CC eluting with CH_2_Cl_2_/MeOH (*v*/*v*, 1:1,) to yield two subfractions (f5.1 and f5.2). Fraction 5.1 (80 mg) was further purified by silica gel CC with CH_2_Cl_2_/MeOH (*v*/*v*, 100:3) to afford compound **6** (6.0 mg) and compound **8** (3.5 mg). Fraction 5.2 (100 mg) was further purified by silica gel CC with CH_2_Cl_2_/MeOH (*v*/*v*, 80:1) to afford compound **7** (12.5 mg).

Compound **1**: C_15_H_15_NO_2_; pale yellow crystals; UV (MeOH) λmax (logε) 231 (3.73), 296 (4.57) nm; IR νmax 3201, 2976, 2220, 1277 cm^−1^; Data of ^1^H (600 MHz, CDCl_3_) and ^13^C NMR (150 MHz, CDCl_3_), see Table 1; HR-ESI-MS: *m*/*z* 242.11756 [M + H]^+^ (calcd. for C_15_H_16_NO_2_, 242.11752).

Compound **2**: C_15_H_17_NO_2_; pale yellow crystals; [α]$\begin{array}{c} 25\\ D \end{array}$+33.5 (c 0.10 MeOH); UV (MeOH) λmax (logε) 353 (4.24), 273 (3.39) nm; Data of ^1^H (600 MHz, CDCl_3_) and ^13^C NMR (150 MHz, CDCl_3_), see Table 2; HR -ESI-MS: *m*/*z* 244.13321 [M + H]^+^ (calcd. for C_15_H_18_NO_2_, 244.13336).

Compound **5**. C_15_H_17_O_5_; white amorphous solid; [α]$\begin{array}{c} 25\\ D \end{array}$−18.8 (c 0.10 MeOH); UV (MeOH) λmax (logε) 243 (3.46), 263 (3.23) nm; ECD (0.11 mM, MeOH) λmax (Δε) 214 (+11.21), 205 (−12.97) nm; Data of ^1^H (600 MHz, CD_3_OD) and _13_C NMR (150 MHz, CD_3_OD), see Table 3; HR-ESI-MS: *m*/*z* 277.1089 [M + H]^−^ (calcd. for C_15_H_17_O_5_, 277.1081).

Compound **6**. C_13_H_15_O_5_; white amorphous solid; [α]$\begin{array}{c} 25\\ D \end{array}$−27.7 (c 0.10 MeOH); UV (MeOH) λmax (logε) 267 (4.28), 292 (3.20) nm; ECD (0.11 mM, MeOH) λmax (Δε) 262 (+53.94), 294 (+50.67), and 332 (−33.65) nm; Data of ^1^H (600 MHz, DMSO-d6) and ^13^C NMR (150 MHz, DMSO-d6), see Table 4; HR-ESI-MS: *m*/*z* 251.0927 [M–H]^−^ (calcd. for C_13_H_15_O_5_, 251.0925).

### 3.4. X-Ray Crystal Data for Compound ***1***, ***2***

The crystals of **1** were obtained through slow volatilization method from CH_2_Cl_2_: MeOH (*v*/*v* 1:1) at room temperature. The crystallographic data were collected on a Rigaku XtaLAB Pro diffractometer with GaKα radiation (λ = 1.34138 Å). The stereo structures of **1** were solved using direct methods with the SHELXS-9729 software package. All non-hydrogen atoms are refined using anisotropic displacement parameters. All hydrogen atoms were located at idealized positions and refined using relative isotropic parameters as riding atoms.

Crystallographic data for **1**: C_15_H_15_NO_2_, f_w_ = 241.28; crystal size = 0.08 × 0.05 × 0.03 mm^3^; T = 150.0 K; monoclinic, space group P2_1_; unit cell parameters: a = 13.1725 (7) Å, b = 6.9213 (4) Å, c = 13.8844 (7) Å, α = 90°, β = 106.550 (2)°, γ = 90°, V = 1213.41 (11) Å^3^, Z = 4, Dcalc = 1.321 g/cm^3^, F (000) = 512.0, μ = 0.453 mm^−1^. A total of 52,206 reflections were collected, which yielded 2991 independent reflections. The final refinement gave R_1_ = 0.0402, wR_2_ = 0.1169 [I ≥ 2σ (I)]. CCDC number: 2357276.

Crystallographic data for **2**: C_15_H_21_NO_4_, f_w_ = 279.33; light-yellow crystal from CH_3_OH; crystal size = 0.13 × 0.12 × 0.1 mm^3^; T = 100.0 (4) K; orthorhombic, space group P2_1_2_1_2_1_; unit cell parameters: a = 6.67510 (10) Å, b = 12.52990 (10) Å, c = 52.8639 (5) Å, α = 90°, β = 90°, γ = 90°, V = 4421.45 (9) Å^3^, Z = 12, Dcalc = 1.259 g/cm^3^, F (000) = 1800.0, μ = 0.747 mm^−1^. A total of 34,035 reflections were collected, which yielded 8754 independent reflections. The final refinement gave R_1_ = 0.0300, wR_2_ = 0.0760 [I ≥ 2σ(I)], Flack parameter = 0.17 (4). CCDC number: 2356318.

### 3.5. ECD Calculation

The conformational searches of the compounds were carried out by means of the Spartan’14 software and with Molecular Merck force field (MMFF) and DFT/TD-DFT calculations. Furthermore, Gaussian 05 program was used to generate and optimize the conformer at B3LYP/3-21G (d) level. Conformers with a Boltzmann distribution of over 1% were chosen for optimization at B3LYP/6-31+G (d, p); meanwhile, ECD calculations were conducted with the TD-DFT method at the B3LYP/6-31+G (d, p) level. The ECD spectra were generated using SpecDis 3.0 (University of Würzburg, Würzburg, Germany) and Origin Pro 8.0 (Origin Lab, Ltd., Northampton, MA, USA) from dipole length rotational strengths by applying Gaussian band shapes with sigma = 0.30 Ev [19].

### 3.6. In Vitro Antimicrobial Activity

The minimum inhibitory concentration (MIC) values of antimicrobial activity of compounds **1**–**4** against three different plant pathogenic bacteria, including *Penicillium italicum, Fusarium oxysporum* and *Colletotrichum musae*, and three human pathogenic bacteria, including *Salmonella typhimurium* (ATCC 6539)*, Escherichia coli* (ATCC 25922) and *Staphylococcus aureus* (ATCC 12598), were determined via serial dilution technique employing 96-well microtiter plates [30,31]. In brief, the bacterial cell suspension was adjusted to an inoculum density of 5 × 10^5^ cfu/mL by comparison with a MacFarland standard before use. The fungal thallus concentration was adjusted to 1 × 10^6^ cfu/mL. The tested compounds in dimethyl sulfoxide (DMSO) were added to PDB/MH broth medium to obtain a series of final concentrations of 100, 50, 25, 12.5, 6.25, 3.13, 1.57, 0.78 μg/mL. DMSO and carbendazim/ciprofloxacin served as negative and positive controls, respectively. The antifungal plates were incubated for 48 h at 27 °C, and the antibacterial plates were incubated for 24 h at 27 °C. Ten microliters of resazurin dissolved in DMSO at 100 μg/mL was added to each well of the microplate, followed by incubation at 27 °C for 3 h. We determined the MIC values by observing color changes using the visual endpoint method. For each parallel experiment, three replicates were measured [18].

### 3.7. Anti-Inflammatory and Cytotoxicity Assays

Cell viability was evaluated using MTT (3-(4,5-Dimethylthiazol-2-yl)-2,5-diphenyltetrazolium bromide) assay [32]. RAW 264.7 cells were seeded in 96-well plates at a density of 5 × 10^3^ cells per well and incubated overnight. Cells were incubated with tested compounds (DMSO as solvent) at 37 °C for 72 h. Subsequently, 50 μL of MTT/medium solution (0.5 mg/mL) was added to each well, and the cells were incubated for 1 h at 37 °C. After removing the MTT/medium, 100 μL of DMSO was added to each well. The plate was shaken to dissolve the precipitate, and activity was measured at 540 nm using a microplate reader. Cell viability was calculated using the following formula: Cell viability/% = (Absorbance of treated cells − Absorbance of blank control)/(Absorbance of negative control − Absorbance of blank control) × 100. The half-maximal inhibitory concentration (IC_50_) was calculated with Origin 8 Pro.

The nitrite concentration in the culture medium was measured using the Griess reagent, as previously reported, with slight modifications. The mouse macrophage cell line RAW 264.7 cells were purchased from the Shanghai Institutes for Biological Sciences and cultured in DMEM (high-glucose) medium supplemented with 10% (*v*/*v*) fetal bovine serum, 100 μg·mL^−1^ penicillin and streptomycin, and 10 mM HEPES at 37 °C in a 5% CO_2_ atmosphere [33]. Cells were pretreated with different samples dissolved in serum-free culture medium containing 0.5% DMSO (10, 5, 2.5, 1.25, and 0.625 μM) for 4 h, followed by stimulation with 1 μg·mL^−1^ LPS for 24 h. Then, 50 μL of cell culture medium was mixed with 100 μL of Griess reagent I and II and incubated at room temperature for 10 min with horizontal shaking, after which the absorbance at 540 nm was measured in a microplate reader. Indomethacin was used as a positive control and was purchased from Sigma-Aldrich (St. Louis, MI, USA) Co. Wells with DMSO were used as a negative control (final DMSO concentration was 0.1%). The NO production inhibition rate was calculated using the flowing formula:

$$\mathrm{NO}\;\mathrm{production}\;\mathrm{inhibition}\;\mathrm{rate}\;(\%)\;=\frac{LPS\;group-Compound\;group}{LPS\;group-DMSO\;group}\times 100$$

IC_50_ is the concentration of the compound that inhibits 50% of NO production relative to the LPS group. All assays were performed in triplicate [34]. The inhibition rates were calculated and plotted versus test concentrations to afford the IC_50_ (±SD) for three independent tests. Data were analyzed using SPSS 26.0 software. *p* < 0.05 was considered statistically significant [35,36].

### 3.8. Molecular Docking

Molecular docking simulations were performed using Autodock tool in AutoDock4.2.6 software [37]. The three-dimensional (3D) crystal structure of iNOS (PDB ID:3E6T) was obtained from the RCSB protein database. Before docking simulations, the original ligands and water molecules in the crystal structures were removed using PyMOL 3.1 software, and the proteins were saved in PDB format (receptor.pdb). The structures of the compounds were drawn using ChemDraw 2D 17.1 software and converted to three-dimensional structures using ChemDraw 3D 17.1 software and then saved as files in PDB format. In addition, the molecular structures were optimized using Gaussian 16 B.01 software. The AutoDock 4.2.6 tool converted the proteins and ligands into PDBQT format for subsequent docking. For proteins, the parameters of the grid box were set to 126 × 126 × 126 points, with a resolution of 0.275 Å, centered at x, y, z = (−20 Å, −5 Å, 15 Å), with a maximum of 2,500,000 assessments, 27,000 generations, a 0.02 gene mutation rate, and a 0.8 cross-over rate, and 100 GA operations were performed using the Lamarckian genetic algorithm (LGA). A tolerance of 2.0 Å was established for each docking’s root-mean-square deviation (RMSD). The binding affinities in terms of binding energy (ΔG in kcal/mol) for the hits and decoys were predicted by both docking engines. The conformer with minimum binding energy, the one with the highest negative value, was chosen for binding interaction analysis. Finally, the results were visualized and analyzed using PyMOL.

## 4. Conclusions

In summary, eight benzofurans, including four new compounds, were identified from the culture broth of *Penicillium crustosum* SCNU-F0046. Their structures were determined based on extensive spectroscopic analysis electronic circular dichroism (ECD) calculations and single-crystal X-ray analysis. All new compounds were tested for antimicrobial activities. The results showed that compound **1** exhibited moderate antibacterial activities against *Salmonella typhimuriumi* and *Staphylococcus aureus,* and compound **6** exhibited potent activities against *Penicillium italicum, Fusarium oxysporum* and *Colletotrichum musae*. In addition, the NO inhibitory activity of compounds **1**–**4** on the LPS-stimulated RWA264.7 cell line was evaluated, and the results indicated that compounds **1** and **4** exhibited potent anti-inflammatory activity with IC_50_ values of 17.3 μM and 16.5 μM, respectively, which is comparable to the positive control (celecoxib) with an IC_50_ of 32.1 μM. This study identified compounds with potential antibacterial and anti-inflammatory activities; the investigation into the mechanisms for their anti-inflammatory and antibacterial properties will be undertaken in our coming work.

## Figures and Tables

**Table 1:** ^1^H (600 MHz) and ^13^C (150 MHz) NMR data for compound **1** (CDCl_3_).

Position	1	
	*δ*_H_ (*J* in Hz)	*δ*_C_, Type
2		163.5, C
3	6.75, s	101.7, CH
3a		149.5, C
4	7.76, d (8.46)	124.8, CH
5	7.51, d (8.46)	120.2, CH
5a		134.8, C
6	7.55, s	119.2, CH
7		151.3, C
9	9.65, s	144.5, CH
9a		115.2, C
9b		124.7, C
10	2.74, s	24.2, CH_3_
11		69.4, C
12	1.78, s	29.0, CH_3_
13	1.78, s	29.0, CH_3_

**Table 2:** ^1^H (600 MHz) and ^13^C (150 MHz) NMR data for compound **2** (CDCl_3_).

Position	2	
	*δ*_H_ (*J* in Hz)	*δ*_C_, Type
2	3, 28, dd (9.84, 9.84)	91.3, CH_2_
3	3.47, m	30.8, CH
3a		122.0, C
4	7.42, d (8.16)	127.8, CH
5	7.08, d (8.16)	120.2, CH
5a		136.6, C
6	7.19, s	118.4, CH
7		155.7, C
9	9.26, s	145.8, CH
9a		113.9, C
9b		150.4, C
10	2.48, s	26.7, CH_3_
11		71.4, C
12	1.29, s	24.8, CH_3_
13	1.54, s	23.6, CH_3_

**Table 3:** ^1^H (600 MHz) and ^13^C (150 MHz) NMR data for compound **5** (CD_3_OD).

Position	5	
	*δ*_H_ (*J* in Hz)	*δ*_C_, Type
2	4.64, t (9.1)	90.5, CH
3	3.25, m	31.6, CH_2_
3a		130.9, C
4	7.17, d (7.8)	125.5, CH
5	7.21, d (7.8)	120.0, CH
5a		135.2, C
6	6.41, d (15.9)	120.6, CH
7	8.08, d (15.9)	143.5, CH
8		170.8, C
9	4.75, s	55.4, CH_2_
9a		121.8, C
9b		160.1, C
10		72.4, C
11	1.33, s	25.7, CH_3_
12	1.24, s	24.8, CH_3_

**Table 4:** ^1^H (600 MHz) and ^13^C (150 MHz) NMR data for compound **6** (DMSO-d6).

Position	6	
	*δ*_H_ (*J* in Hz)	*δ*_C_, Type
2		107.7, C
3		199.7, C
3a		118.7, C
4	7.47, d (7.9)	122.4, CH
5	7.25, d (7.9)	112.0, CH
5a		153.7, C
6	4.74, m	60.3, CH_2_
7	4.56, m	52.5, CH_2_
8		122.8, C
8a		168.8, C
9	2.07, m	33.5, CH
10	0.80, d (6.8)	15.9, CH_3_
11	0.99, d (6.8)	15.6, CH_3_
OH-2	7.55, s	
OH-6	5.33, t (5.6)	
OH-7	5.00, t (5.2)	

**Table 5:** Antimicrobial activities of compounds **1**–**2** and **5**–**6** ^a^.

Compound	*Salmonella typhimurium*	*Escherichia coli*	*Staphylococcus aureus*	*Penicillium italicum*	*Fusarium* *oxysporum*	*Colletotrichum musae*
**1**	12.5	25	12.5	50	50	50
**2**	50	50	25	50	50	25
**5**	50	50	50	12.5	50	25
**6**	50	100	50	12.5	50	12.5
Carbendazim ^b^	-	-	-	0.78	1.56	0.78
Ciprofloxacin ^c^	0.2	0.8	0.1	-	-	-

^a^ minimum inhibitory concentration values (MIC, μg/mL); data are shown as the means from three parallel experiments. ^b,c^ positive control.

**Table 6:** Anti-inflammatory activity of compounds **1**–**4**.

Compound	Inhibition of NO Production (IC_50_/μM) ^b^	Cytotoxicity (CC_50_/μM) ^b^
**1**	17.3 ± 1.6	>50
**2**	31.5 ± 2.3	>50
**3**	42.8 ± 4.7	>50
**4**	16.5 ± 3.4	>50
Celecoxib ^a^	32.1 ± 1.7	>50

^a^ positive control. ^b^ IC_50_ values are taken as means ± standard deviation from three independent experiments.; data are shown as the means from three parallel experiments.

## Data Availability

The authors confirm that the data supporting the findings of this study are available within the article.

## Supplementary Materials

The following supporting information can be downloaded at: https://www.mdpi.com/article/10.3390/ijms26167861/s1.
