# Supplementary material for: Benzofuran Derivatives with Antimicrobial and Anti-Inflammatory Activities from Penicillium crustosum SCNU-F0046
Source: Int J Mol Sci. 2025 Aug 14;26(16):7861. doi: 10.3390/ijms26167861 (PMC12386745; doi:10.3390/ijms26167861)
Supplement: Supplementary file 1 [file ijms-26-07861-s001.zip › ijms-3794672-supplementary.pdf]

Supporting information

Benzofuran derivatives with antimicrobial and anti-inflammatory activities from *Pencillium crustosum* SCNU-F0046

Chen Chen, Jinbi Kang, Ruiqi Zhang, Hao Jia, Zirong Lin, Zhengming Liu, Rongrong Liu, Xinyi Zou, Yuhua Long *

Guangzhou Key laboratory of Analytical Chemistry for Biomedicine, School of Chemistry, South China Normal University, Guangzhou 510006, China; chenchen2021@m.scnu.edu.cn (C.C.); 2024022687@m.scnu.edu.cn (J.K.); rickie@m.scnu.edu.cn (R.Z); haojia@m.scnu.edu.cn (H. J.); linzirong@m.scnu.edu.cn (Z. L.); 2023022616@m.scnu.edu.cn (Z.L.); rongrongliu@m.scnu.edu.cn (R.L.); 2024022764@m.scnu.edu.cn (X.Z.)

***** Correspondence: longyh@scnu.edu.cn

Content

Figure S1 HRESIMS of compound **1**
Figure S2 ^{1}H NMR spectrum (600 MHz, chloroform -*d*)) of compound **1**
Figure S3 ^{13}C NMR spectrum (150 MHz, chloroform -*d*)) of compound **1**
Figure S4 HSQC spectrum (600 MHz, chloroform -*d*)) of compound **1**
Figure S5 HMBC spectrum (600 MHz, chloroform -*d*)) of compound **1**
Figure S6 ^{1}H-^{1}H COSY spectrum (600 MHz, chloroform -*d*)) of compound **1**
Figure S7 HRESIMS of compound **2**
Figure S8 ^{1}H NMR spectrum (600 MHz, chloroform -*d*) of compound **2**
Figure S9 ^{13}C NMR spectrum (150 MHz, chloroform -*d*) of compound 2
Figure S10 HSQC spectrum (600 MHz, chloroform -*d*) of compound **2**
Figure S11 HMBC spectrum (600 MHz, chloroform -*d*) of compound **2**
Figure S12 ^{1}H-^{1}H COSY spectrum (600 MHz, chloroform -*d*) of compound **2**
Figure S13 HRESIMS of compound **5**
Figure S14 ^{1}H NMR spectrum (600 MHz, methanol-d_4) of compound **5**
Figure S15 ^{13}C NMR spectrum (150 MHz, methanol-d_4) of compound **5**
Figure S16 HMQC spectrum (600 MHz, methanol-d_4) of compound **5**
Figure S17 HMBC spectrum (600 MHz, methanol-d_4) of compound **5**
Figure S18 ^{1}H-^{1}H COSY spectrum (600 MHz, methanol-d_4) of compound **5**
Figure S19 HRESIMS of compound **6**
Figure S20 ^{1}H NMR spectrum (600 MHz, DMSO-d_6) of compound **6**
Figure S21 ^{13}C NMR spectrum (150 MHz, DMSO-d_6) of compound **6**
Figure S22 HMQC spectrum (600 MHz, DMSO-d_6) of compound **6**
Figure S23 HMBC spectrum (600 MHz, DMSO-d_6) of compound **6**
Figure S24 ^{1}H-^{1}H COSY spectrum (600 MHz, DMSO-d_6) of compound **6**

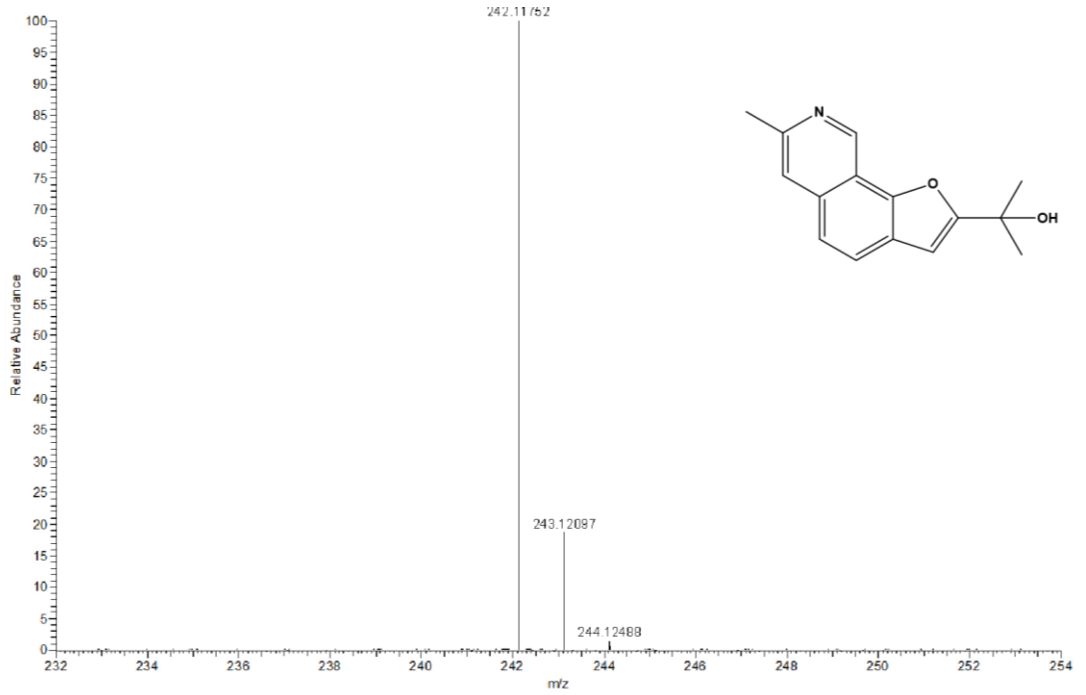

SPECTRUM - simulation:

m/z	Theo. Mass	Delta (ppm)	RDB equiv.	Composition
242.11752	242.11756	-0.15	8.5	C15 H16 O2 N

Figure S1 HRESIMS of compound **1**

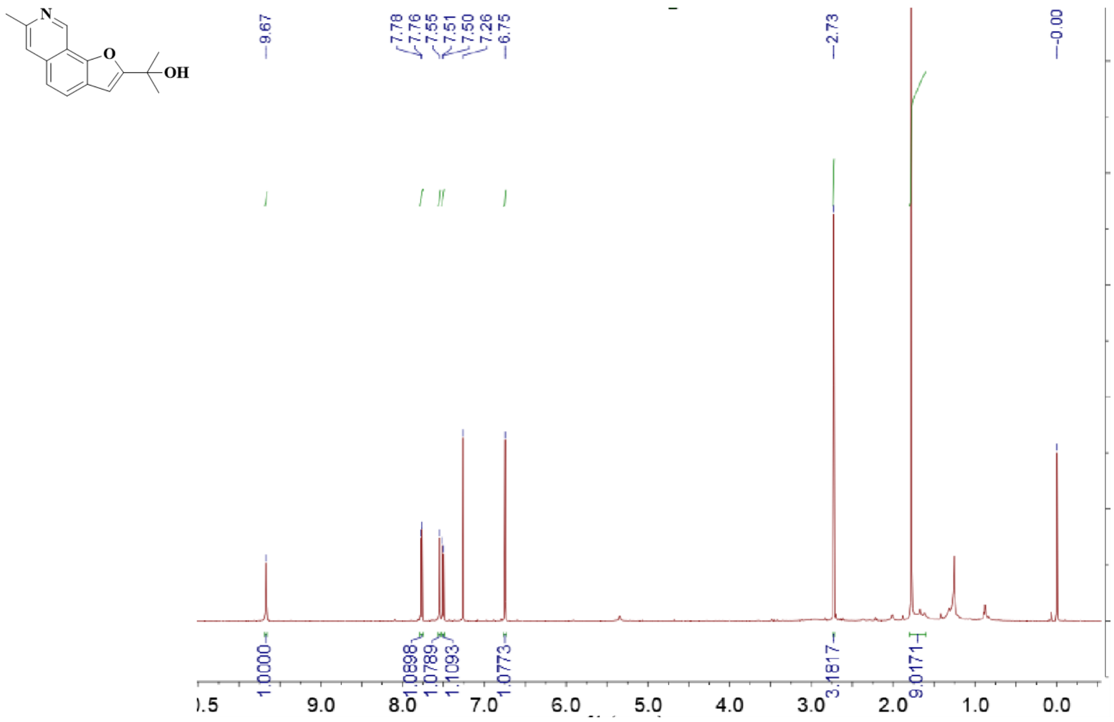

Figure S2 ^{1}H NMR spectrum (600 MHz, chloroform -*d*)) of compound **1**

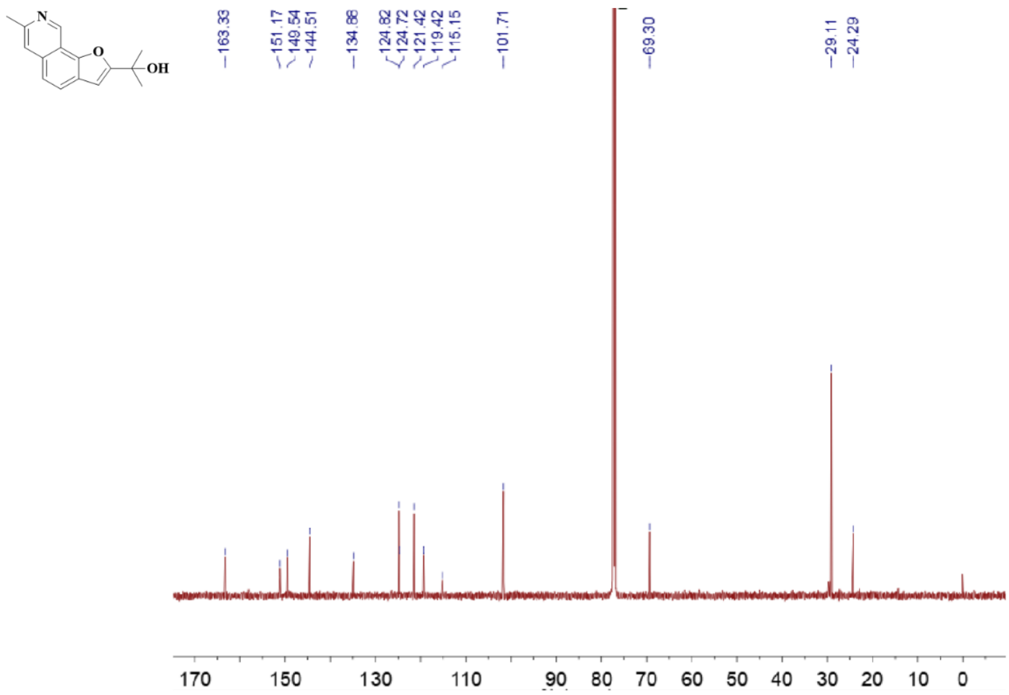

Figure S3 ^{13}C NMR spectrum (150 MHz, chloroform -*d*)) of compound **1**

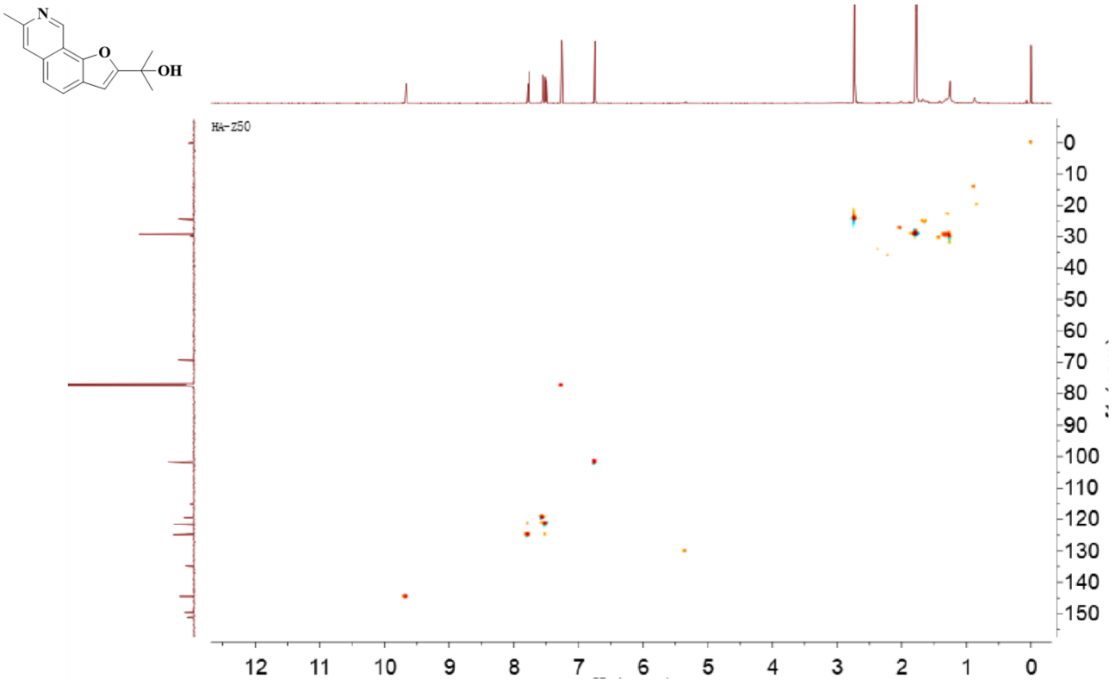

Figure S4 HMQC spectrum (600 MHz, chloroform -*d*)) of compound **1**

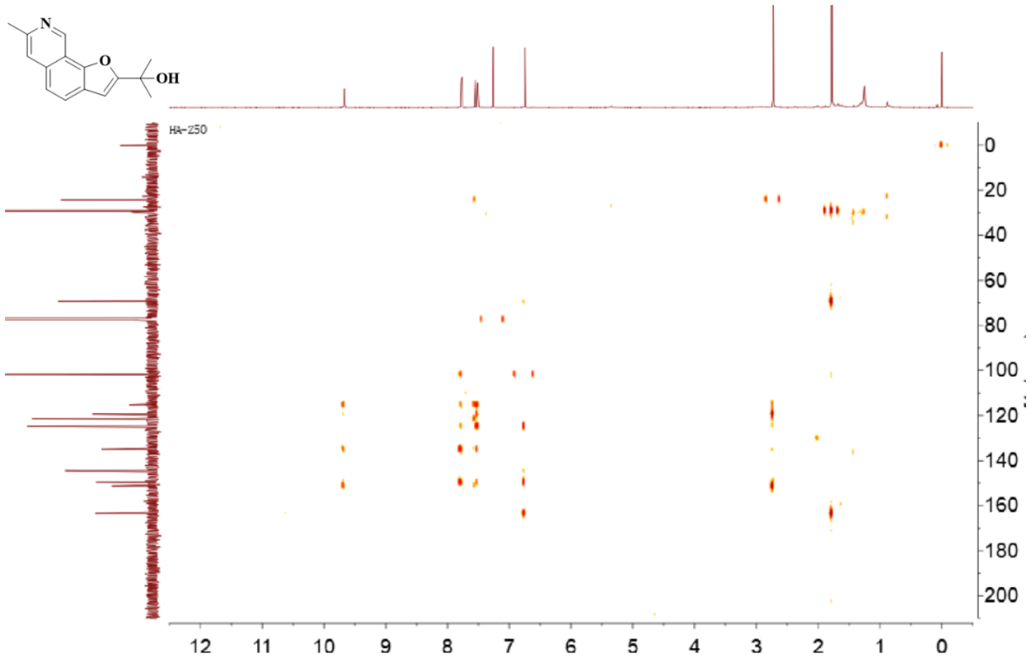

Figure S5 HMBC spectrum (600 MHz, chloroform -*d*)) of compound **1**

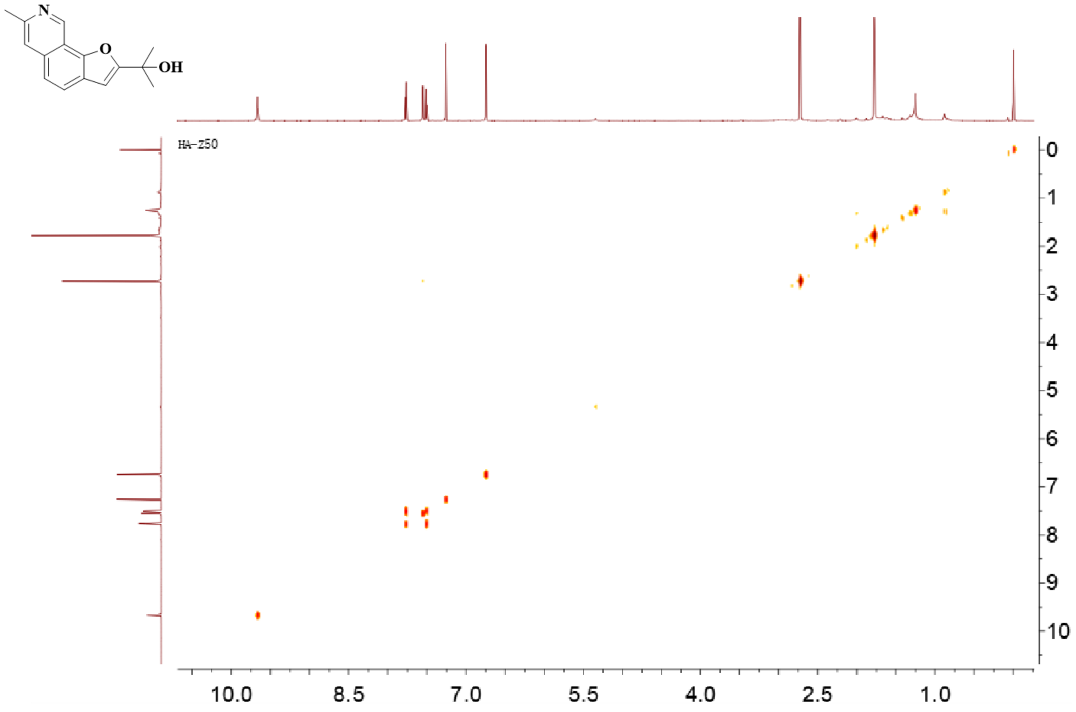

Figure S6 ^{1}H-^{1}H COSY spectrum (600 MHz, chloroform -*d*)) of compound **1**

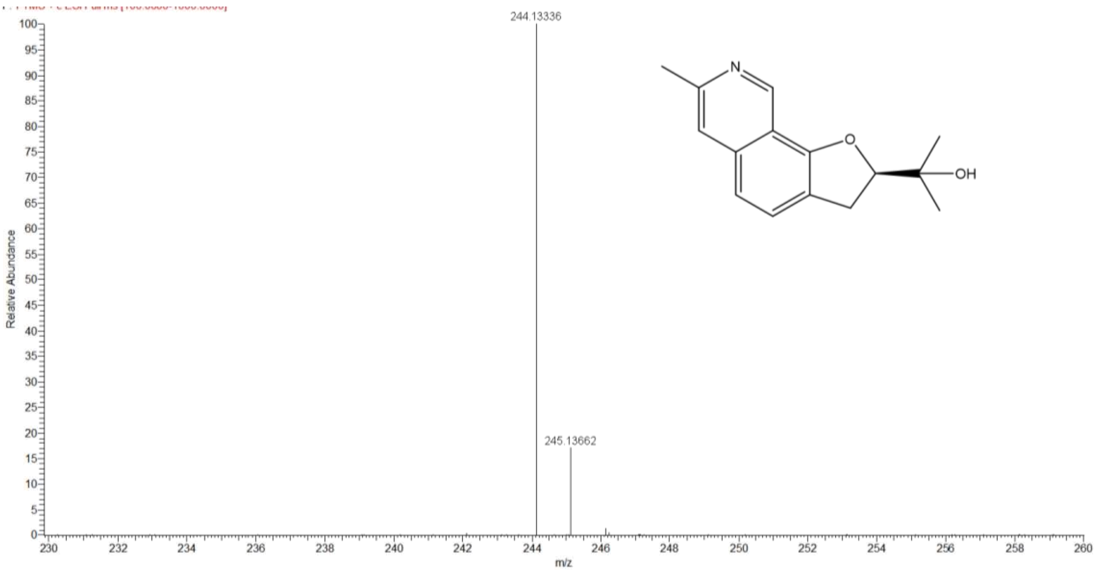

SPECTRUM - simulation:				
m/z	Theo. Mass	Delta (ppm)	RDB equiv.	Composition
244.13336	244.13321	0.63	7.5	C15 H18 O2 N

Figure S7 HRESIMS of compound **2**

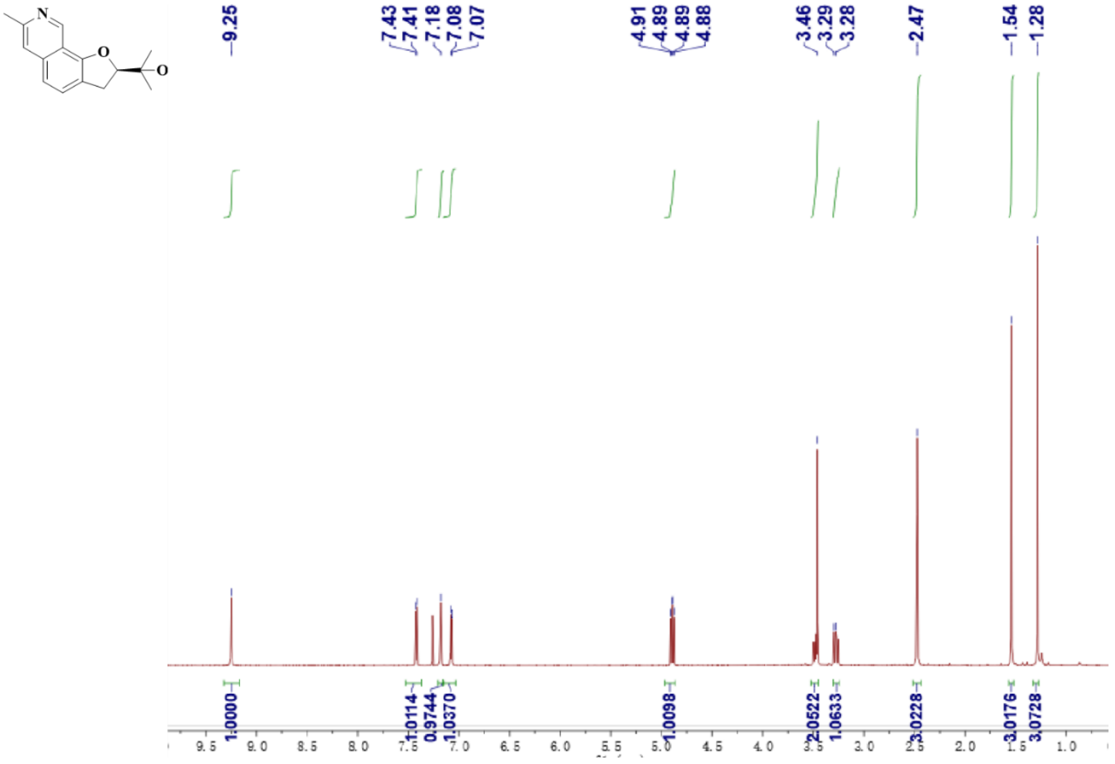

Figure S8 ^{1}H NMR spectrum (600 MHz, chloroform -*d*) of compound **2**

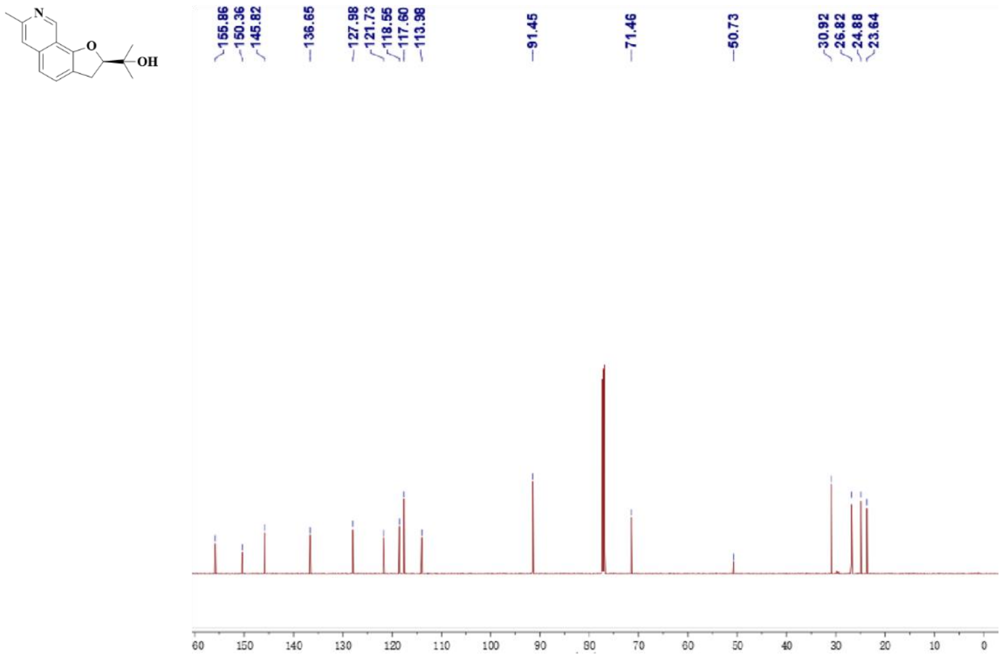

Figure S9 ^{13}C NMR spectrum (150 MHz, chloroform -*d*) of compound **2**

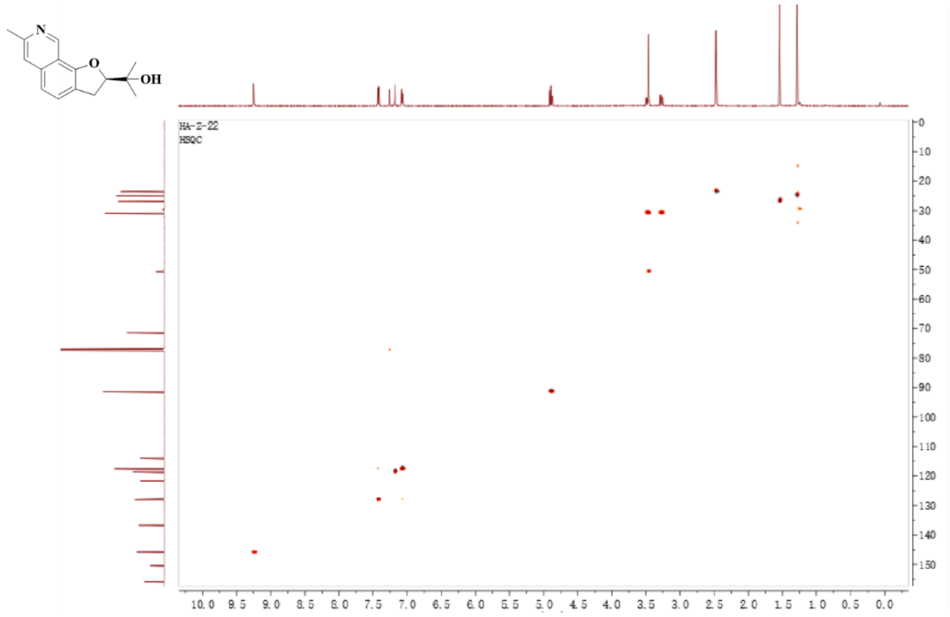

Figure S10 HMQC spectrum (600 MHz, chloroform -*d*) of compound **2**

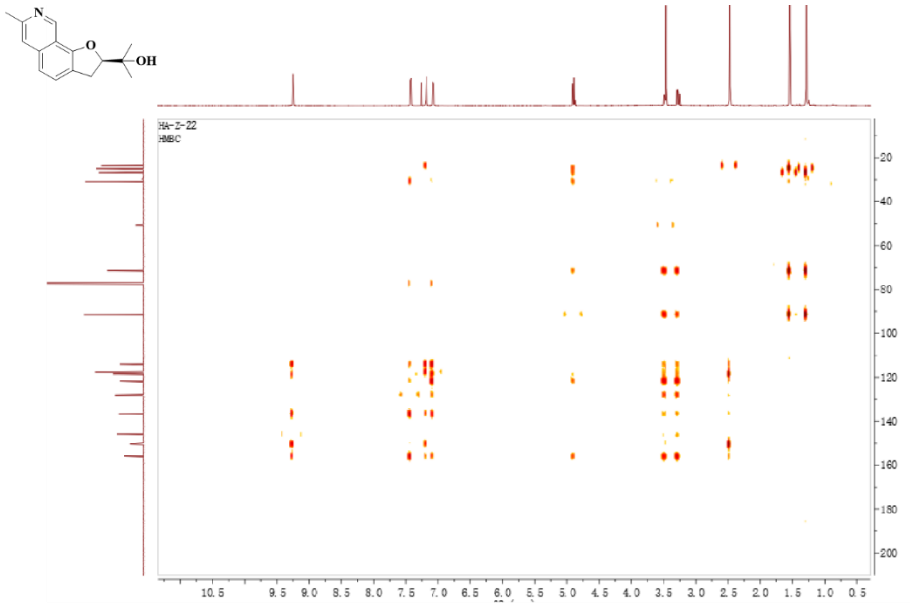

Figure S11 HMBC spectrum (600 MHz, chloroform -*d*) of compound **2**

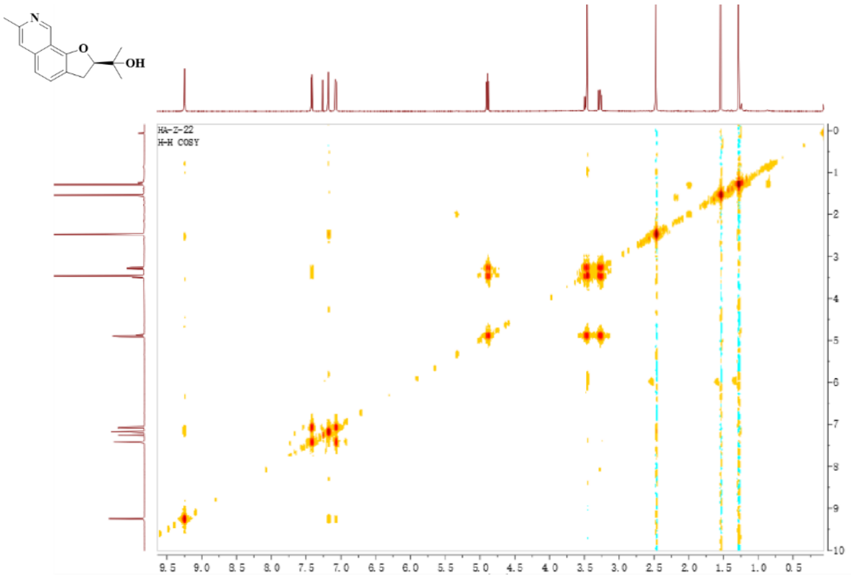

Figure S12 ^{1}H-^{1}H COSY spectrum (600 MHz, chloroform -*d*) of compound **2**

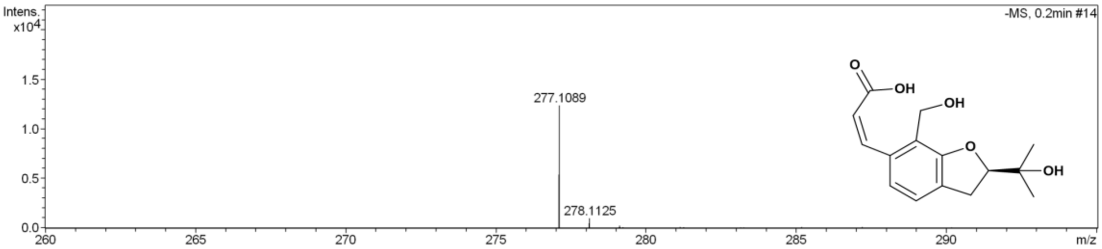

Meas. m/z	Ion Formula	m/z	z	err [ppm]	mSigma	rdb	e⁻Conf	N-Rule

277.1089	$C_{15}H_{17}O_5$	277.1081	1-	-2.9	51.1	7.5	even	ok

Figure S13 HRESIMS of compound **5**

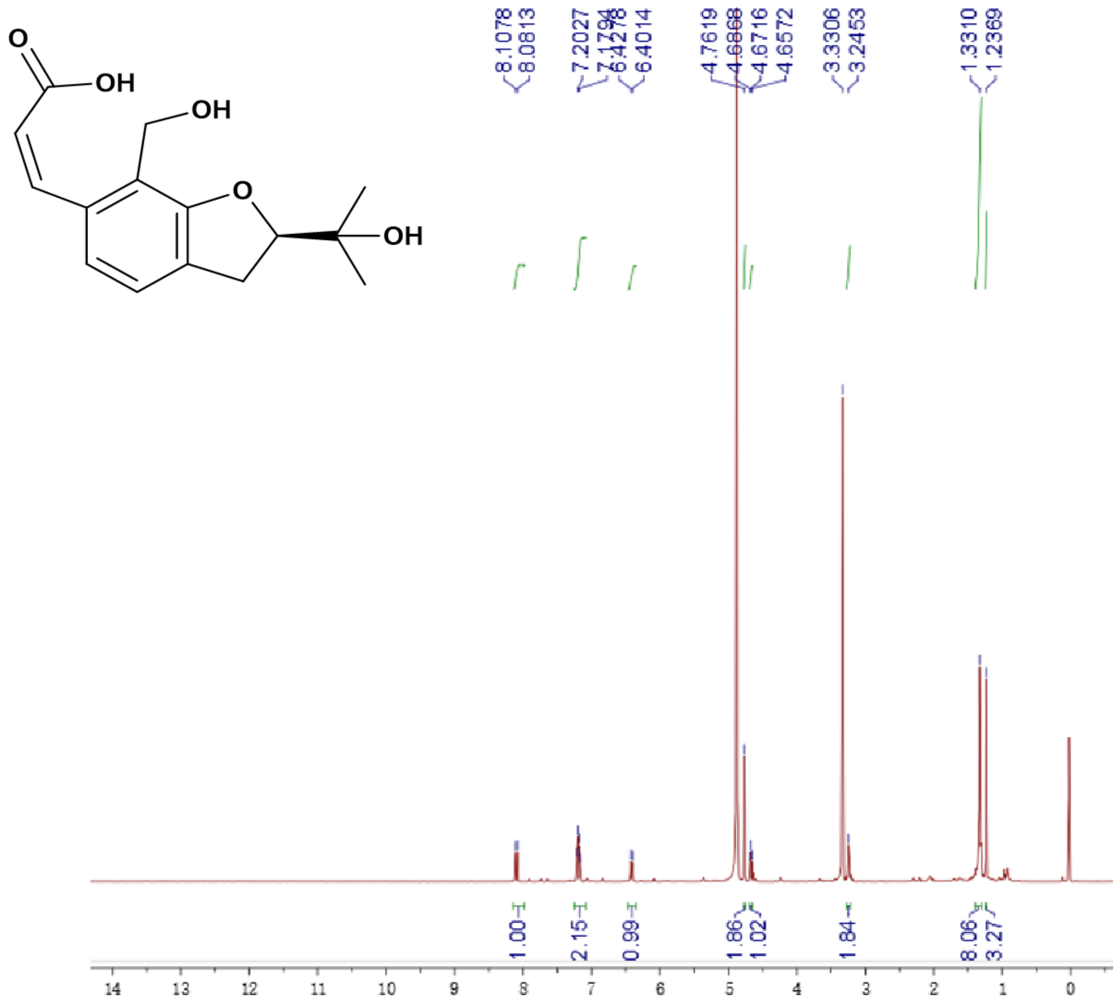

Figure S14 ^{1}H NMR spectrum (600 MHz, methanol-d_4) of compound **5**

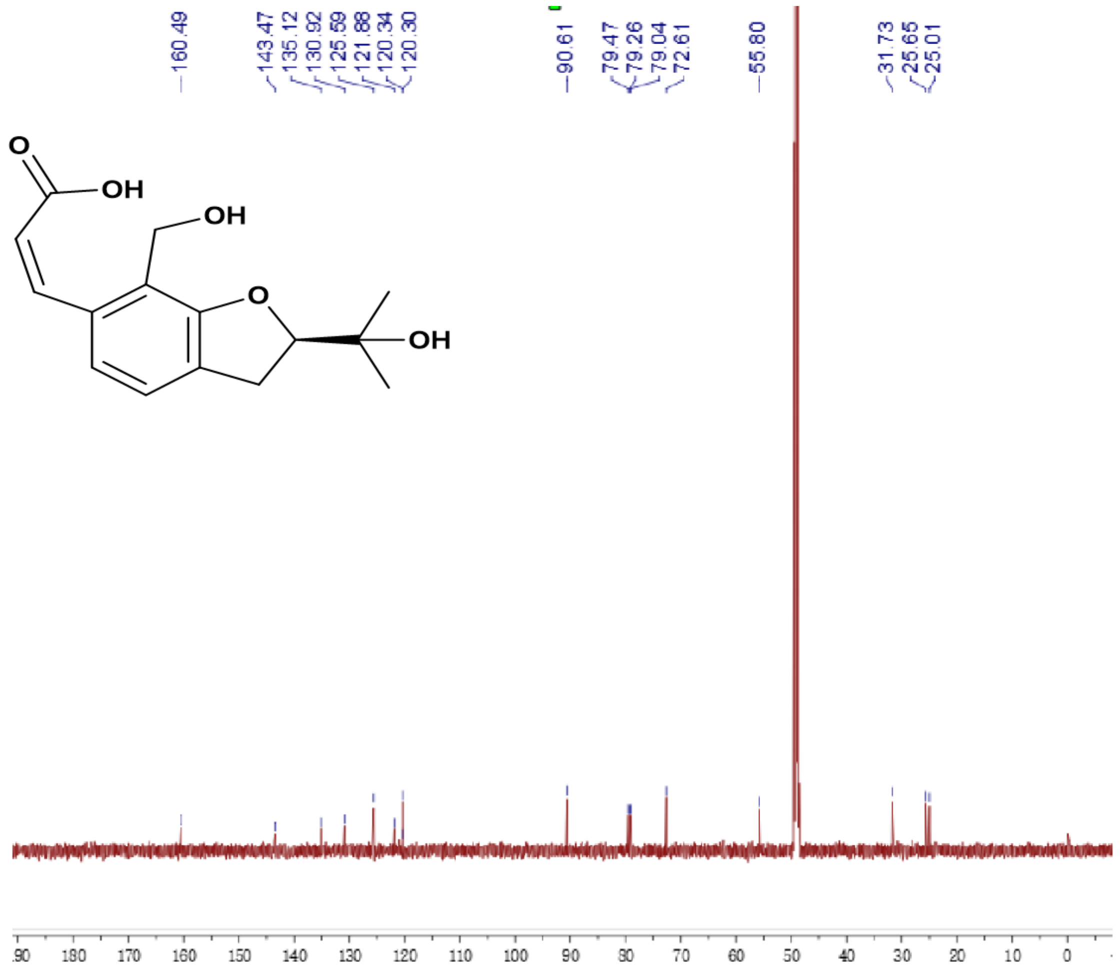

Figure S15 ^{13}C NMR spectrum (150 MHz, methanol-d_4) of compound **5**

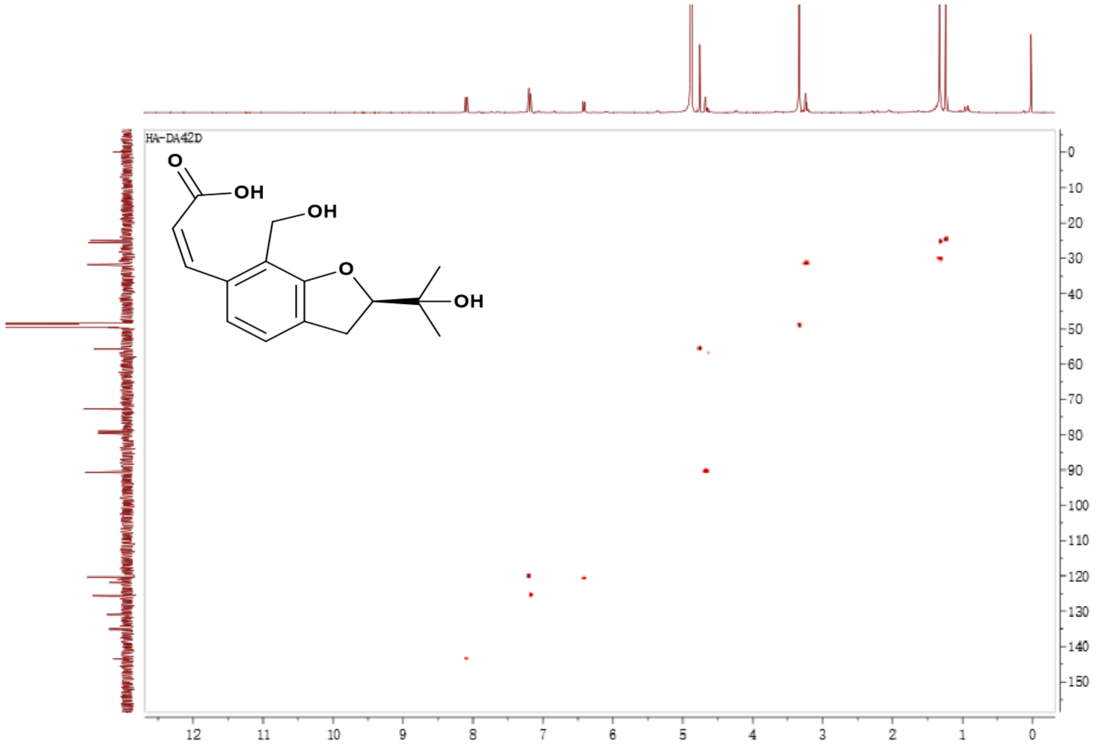

Figure S16 HMQC spectrum (600 MHz, methanol-d_4) of compound **5**

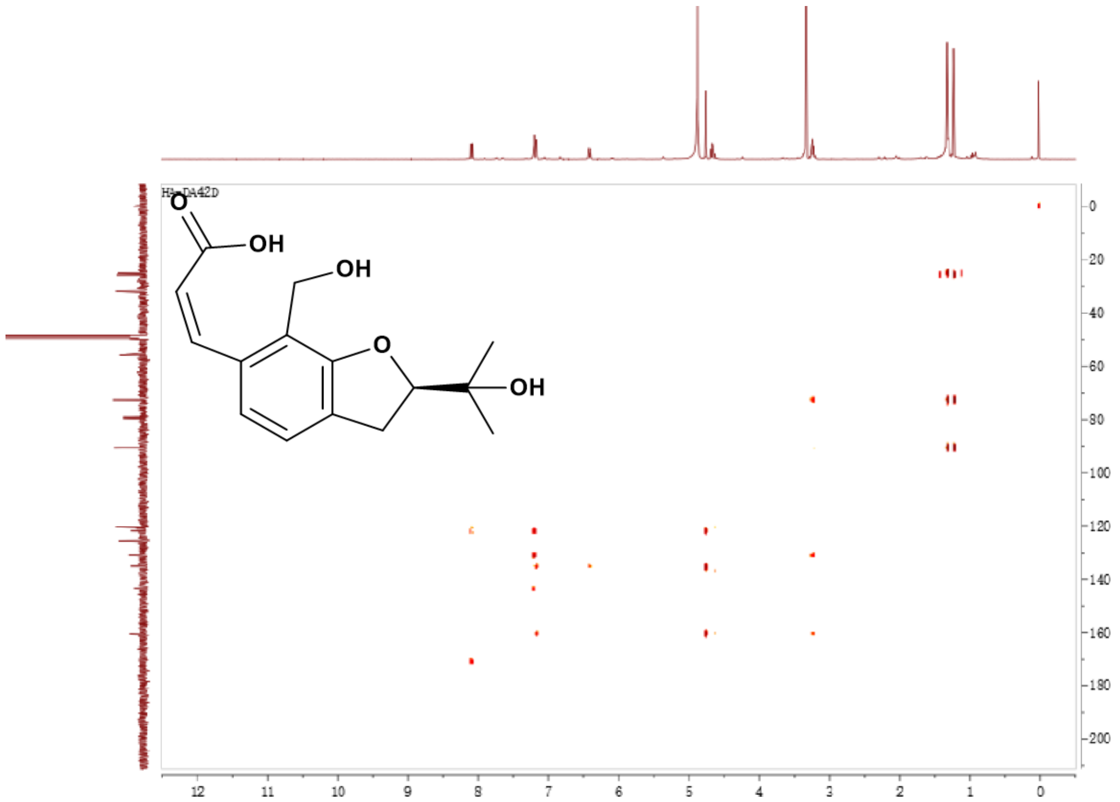

Figure S17 HMBC spectrum (600 MHz, methanol-d_4) of compound **5**

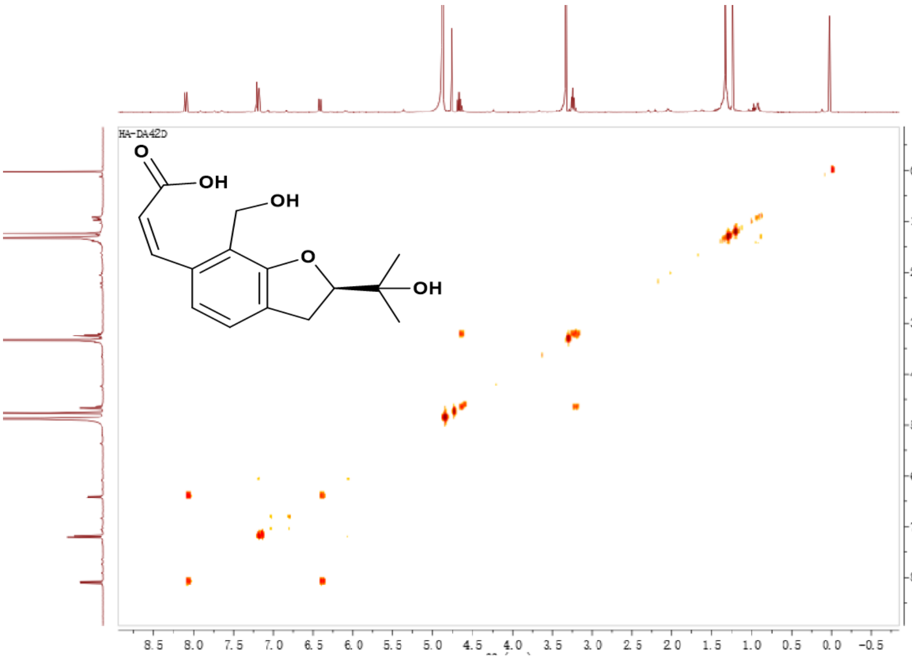

Figure S18 ^{1}H-^{1}H COSY spectrum (600 MHz, methanol-d_4) of compound **5**

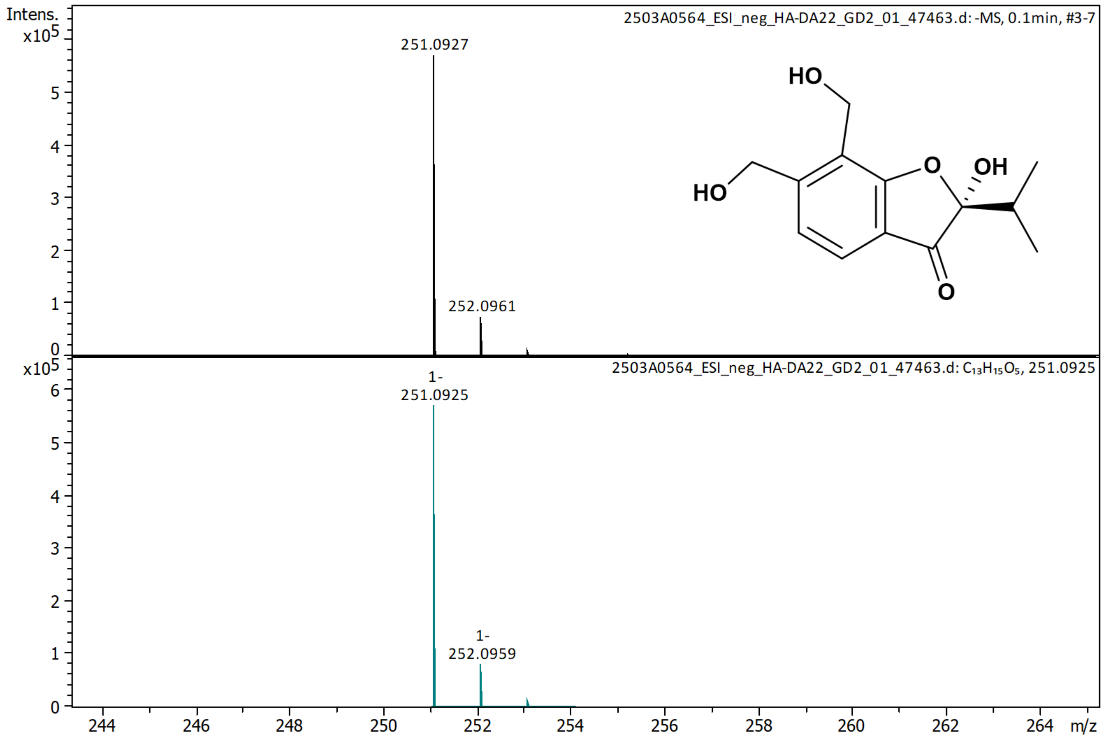

Meas. m/z	Ion Formula	m/z	z	err [ppm]	mSigma	rdb	e⁻ Conf	N-Rule
251.0927	$C_{13}H_{15}O_5$	251.0925	1-	-0.6	5.2	6	even	ok

Figure S19 HRESIMS of compound **6**

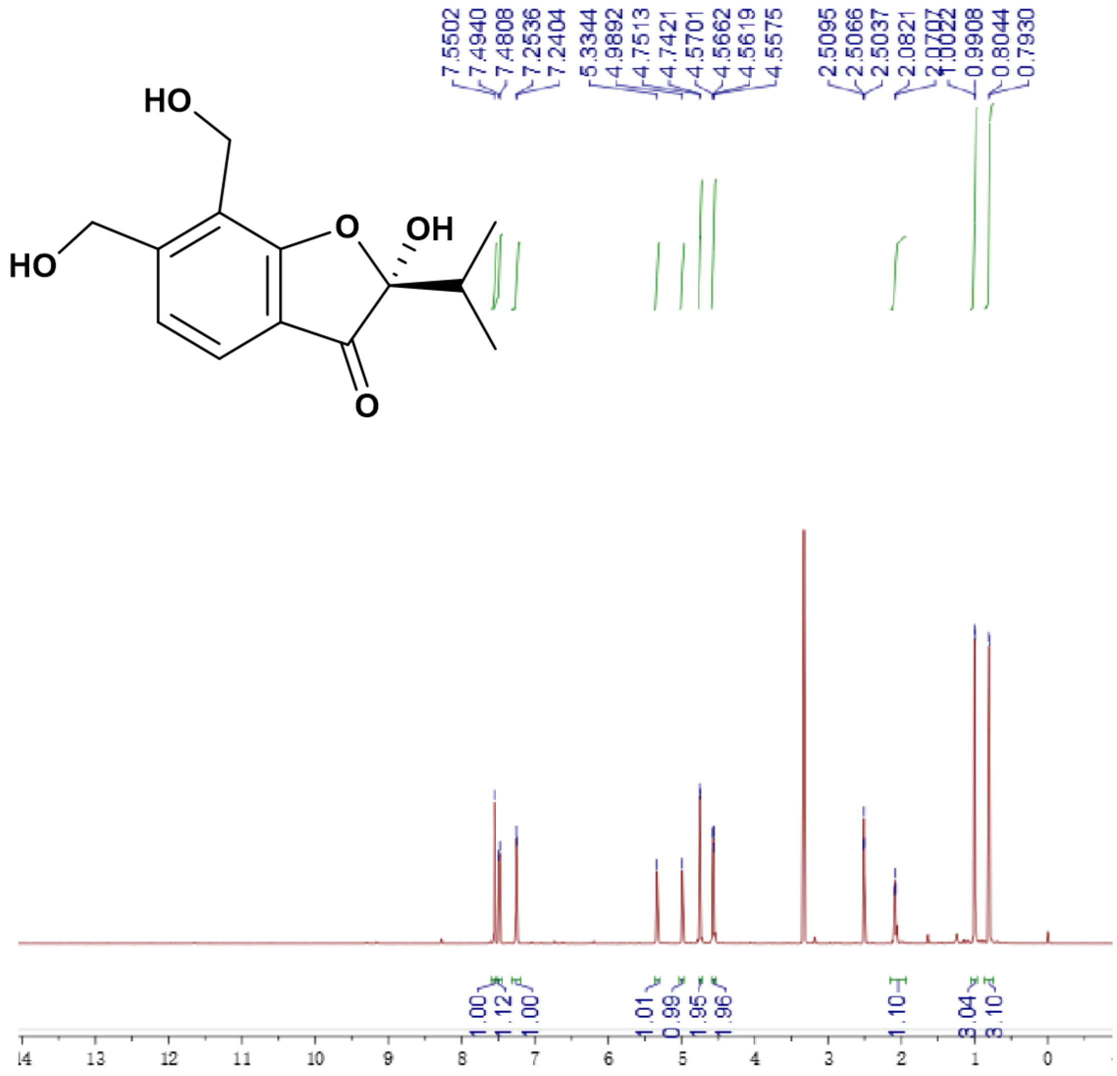

Figure S20 ^{1}H NMR spectrum (600 MHz, DMSO-d_6) of compound **6**

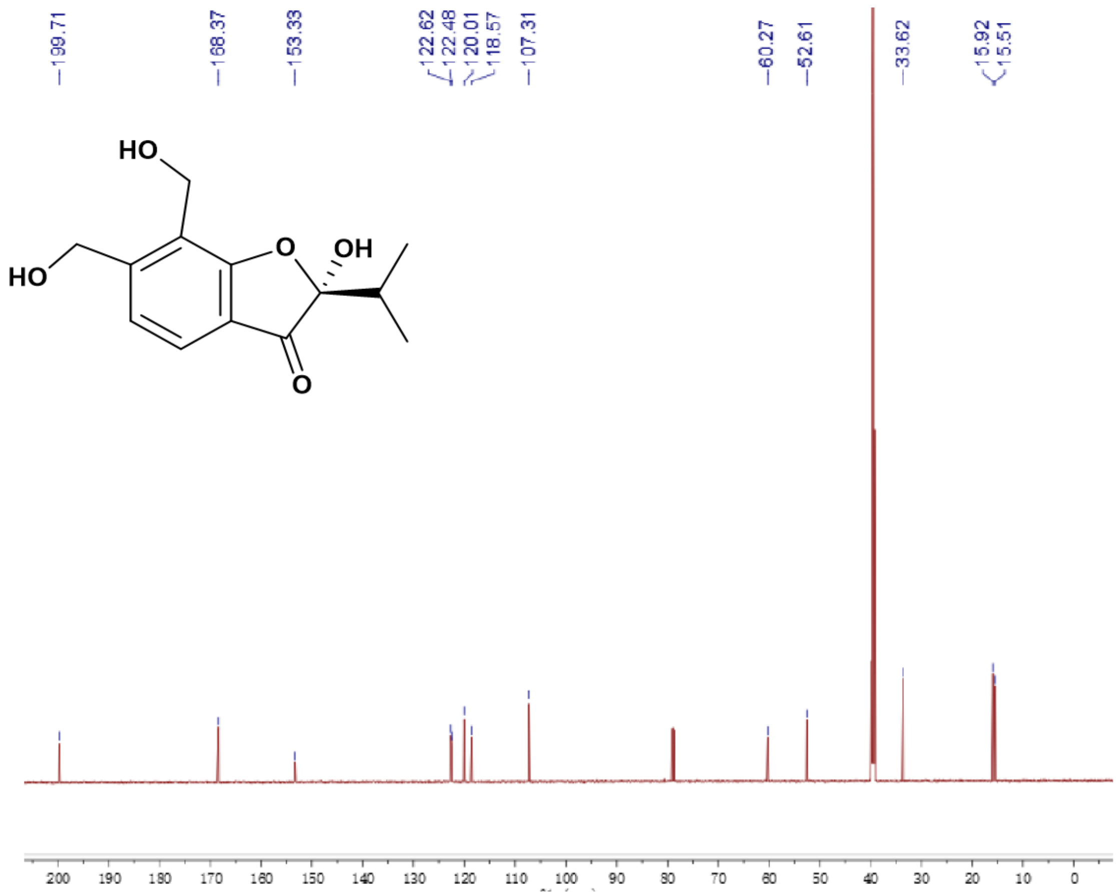

Figure S21 ^{13}C NMR spectrum (150 MHz, DMSO-d_6) of compound **6**

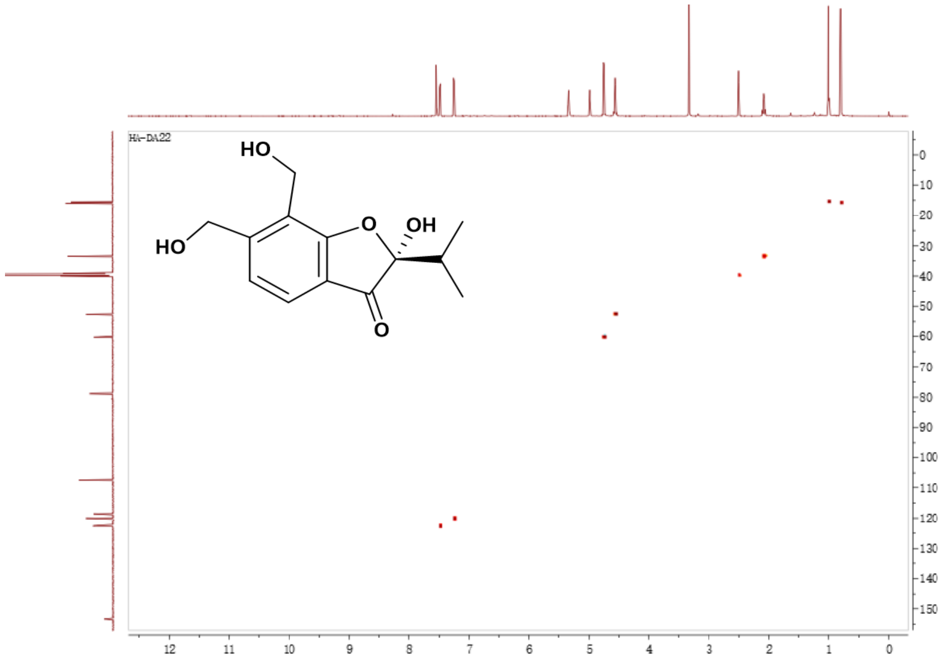

Figure S22 HMQC spectrum (600 MHz, DMSO-d_6) of compound **6**

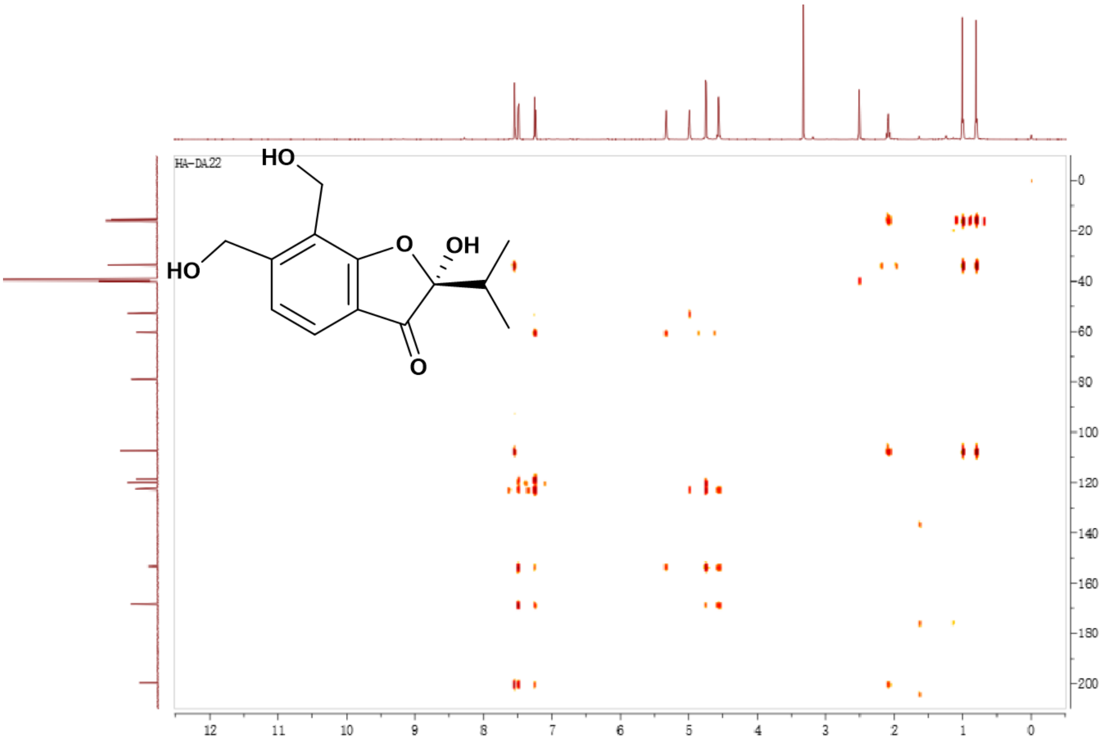

Figure S23 HMBC spectrum (600 MHz, DMSO-d_6) of compound **6**

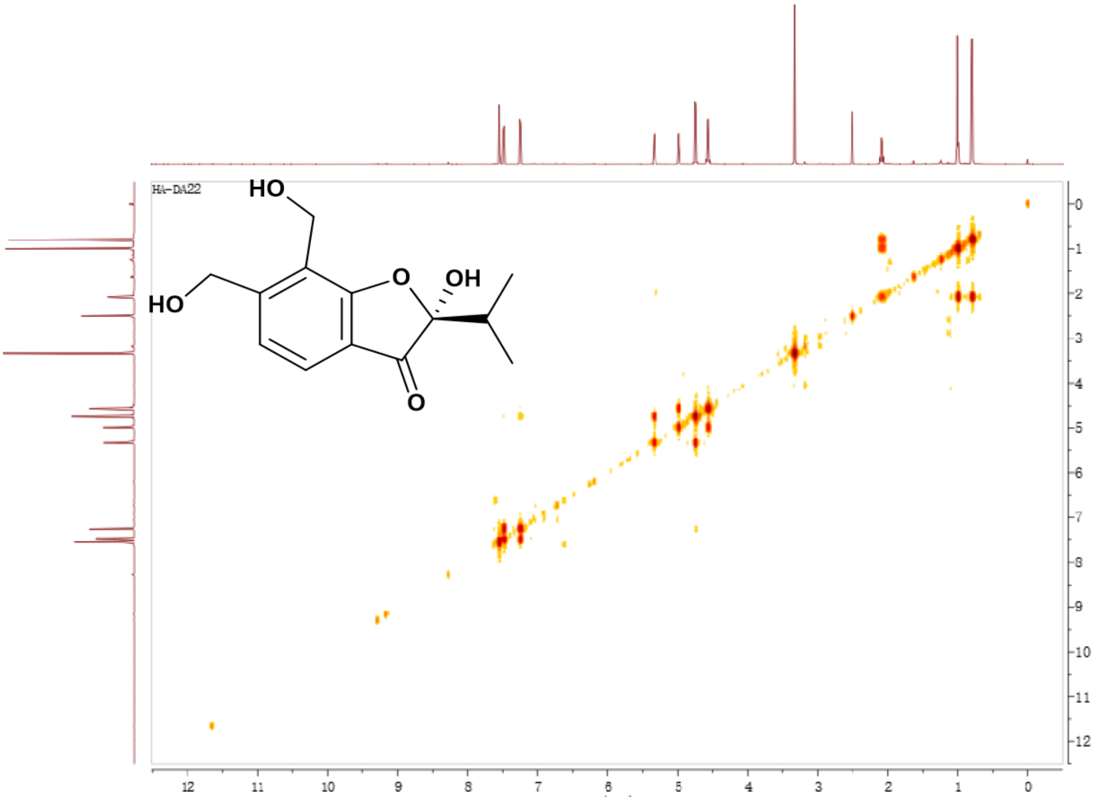

Figure S24 ^{1}H-^{1}H COSY spectrum (600 MHz, DMSO-d_6) of compound **6**
